# Full thermo-mechanical coupling using eXtended finite element method in quasi-transient crack propagation

**DOI:** 10.1186/s40323-018-0112-9

**Published:** 2018-07-03

**Authors:** Fakhreddine Habib, Luca Sorelli, Mario Fafard

**Affiliations:** 1 Aluminium Research Centre-REGAL and Department of Civil and Water Engineering, Laval University, 1065 avenue de la medecine, Quebec, QC G1V 0A6 Canada; 2 Center for Research on Concrete Infrastructure-CRIB and Department of Civil and Water Engineering, Laval University, 1065 avenue de la medecine, Quebec, QC G1V 0A6 Canada

**Keywords:** Thermo-mechanical, Extended finite element method, Full coupling, Crack growth, Stress intensity factors computation, Quasi-transient

## Abstract

This work aims to present a complete full coupling eXtended finite element formulation of the thermo-mechanical problem of cracked bodies. The basic concept of the extended finite element method is discussed in the context of mechanical and thermal discontinuities. Benchmarks are presented to validate at the same time the implementation of stress intensity factors and numerical mechanical and thermal responses. A quasi-transient crack propagation model, subjected to transient thermal load combined with a quasi-static crack growth was presented and implemented into a home-made object-oriented code. The developed eXtended finite element tool for modeling two-dimensional thermo-mechanical problem involving multiple cracks and defects are confirmed through selected examples by estimating the stress intensity factors with remarkable accuracy and robustness.

## Background

The interest in fracture mechanics and its applications has gained considerable importance in recent years in various industries: aerospace engineering, automobile industry, civil engineering, etc. This attention is due to the high cost caused by the presence of cracks and defects, which require more energy, time, substantial efforts and dedicated strategies regarding intervention, maintenance, repair, etc. Practically, taking into account the real environmental conditions in service has become essential, when the material is subject to a significant gradient of temperature. For instance, temperature change in real structures, where the deformation are constrained, can engender a mechanical load and a high-stress concentration around crack tips. Subsequently, crack can propagate with a, *a priori*, not known orientation, direction, intensity etc. Since, cracks cannot be eliminated under any circumstances; this prompts engineers to guide our efforts towards winning strategies in prevention, design and especially analysis that can be provided by the tool of numerical modeling.

The numerical modeling of cracked domains using finite element method (FEM) has clearly stood aside for the eXtended finite element method (XFEM) in the last two decades. XFEM has been able to provide essential answers for several situations, where the FEM method becomes numerically very expensive to have an optimal convergence, such as singularities, strong discontinuities, high gradient, moving surfaces, etc. This technique allows, by prior knowledge of the physical behavior of the problem, to enrich the space of the solutions by non-polynomial asymptotic functions when it is a singularity and a jump-function when it comes to a discontinuity or a combination of both of them. The resulting approximation space has to reproduce the Partition of Unity (PU), Babuška and Melenk [1]. The first work that introduced enriched FEM was Belytchko and Black’s paper [2] which presented an implicit description of the crack with minimal remeshing. Moës et al. [3] improved this technique by incorporating a more suitable way to consider the discontinuities throughout the crack faces away from the crack tip by the generalized Heaviside function and branching functions for the near crack tip. Daux et al. [4] later extend the approach for multiple cracks and holes for the mechanical problem.

Sukumar et al. [5] used the XFEM to model fracture in three-dimensional by using the PU concept, where the two-dimensional asymptotic crack tip displacement fields were added to the FE approximation to account for the crack. The XFEM for non-planar cracks in three dimensions illustrating the crack geometry using two signed distance functions was presented by Moës et al. [6]. Sukumar and Prévost [7] extended XFEM for two-dimensional crack modeling in isotropic and bimaterial media and later to demonstrate the numerical modeling of stress intensity factors in crack growth problems in Sukumar, and Prévost [8]. Lee et al. [9] exposed a combination of the XFEM and the mesh superposition method for modeling of stationary and growing cracks, where a step function implicitly described the discontinuity on the PU, and the crack tip was modeled by superimposed quarter point elements on an overlaid mesh. Budyn et al. [10] displayed a model for multiple crack growth considering the junction of cracks in brittle materials using XFEM, which does not require remeshing as the cracks grow.

Other XFEM aspects have been addressed: In contact, Khoei et al. [11] used XFEM to model the frictional contact problem using the penalty method. Nistor et al. [12] developed an approach to couple the XFEM with the Lagrangian large sliding frictionless contact algorithm. An algorithm based on node-to-segment XFEM contact was presented by Khoei et al. [13] based on the XFEM to model the large deformation-large sliding contact problem using the penalty approach. In stabilization aspect, an XFEM pre-conditioner which stabilizes the enrichments by applying Cholesky decompositions to certain sub-matrices of the stiffness matrix was proposed by Béchet et al. [14]. Menk et al. [15] expose another pre-conditioning method suited for parallel computation. Also, another approach initially developed by Hansbo et al. [16] to simulate strong and weak discontinuities in solid mechanics. A similar method was used by Song et al. [17], named phantom nodes, for shear modeling dynamic crack and shear band propagation. Rabczuk et al. [18] developed a new crack tip element for the phantom node method suited for one-point quadrature scheme and can be used with other general quadrature schemes. XFEM numerical integration aspect is performed by Dolbow et al. [19] by using a sub-triangulation for computing the element area below and above the crack and to set criteria for node enrichment with discontinuity function. Laborde et al. [20] used a singular mapping for each sub-triangle and a bidirectional Gauss quadrature in each direction. In Ventura [21], the constructing sub-cells in the numerical integration of discontinuity functions is removed by defining an equivalent polynomial function. Schwarz–Christoffel conformal mapping was used to map an arbitrary polygon onto a unit disk by Natarajan et al. [22]. A fairly comprehensive review of the different aspects of XFEM was presented by Khoei [23]. All these advances in XFEM mentioned before are in the field of solid mechanics.

In this paper, the approach taken is based on a semi-implicit thermo-mechanical-crack-growth algorithm in which the combined full coupling thermal and mechanical responses have to be estimated beforehand. Then, the developed numerical fracture mechanics module takes those responses as inputs to evaluate the stress intensity factors, J-integral, the update of the crack in growth, etc. This actualization is done by an implicit description of the crack, using the Level-Set Method (LSM) presented firstly by Osher and Sethian [24]. The LSM provides a fundamental complementary to know when, where and how to enrich the crack by determining its relative position. Stolarska et al. [25] introduced an algorithm that combines the XFEM and LSM to model mechanical crack growth, where the LSM was used to model the crack surface and crack tip locations. Moreover, stress intensity factors (SIFs) computation, as the prime parameter of prediction, makes it possible to obtain an essential knowledge of the behavior of the crack. This evaluation enables to predict whether the structure becomes unsafe in service conditions, especially when it is in a thermo-mechanical context, where the spatial distribution of the mechanical stresses induced by the thermal field is unpredictable.

Interest in thermo-mechanical applications appeared later with Michlik and Berndt [26] presented an approach of thermo-mechanical XFEM analysis to account for the existence of cracks in thermal barrier coating for predicting an effective thermal conductivity and Young’s moduli of multi-layered. Duflot [27] used the XFEM for the analysis of steady-state thermally stressed, cracked solids in thermo-elastic problems, where he enriched both thermal and mechanical fields to represent the discontinuous temperature and displacement. Fagerstöm and Larsson [28] presented a thermo-mechanical fracture formulation based on discontinuous representation for temperature and displacements fields applicable to the fracture process zone into a cohesive zone. Zamani et al. [29] proposed a higher order XFEM to predict the SIFs for thermo-elastic with stationary cracks, The computation of SIF is extracted directly from the XFEM degrees of freedom. Zamani et al. [30], in a later work, implemented the XFEM to model the effect of the mechanical and thermal shocks on a body with a stationary crack. Lee et al. [31] presented an XFEM method for the analysis of heat conduction at submicron scales of geometrically complex nanostructured heterogeneous materials. Fan et al. [32] used XFEM to investigate the effect of thermally grown oxide on multiple surfaces cracking behavior in an air plasma sprayed thermal barrier coating system. Hosseini et al. [33] introduced a computational method based on the XFEM for fracture analysis of isotropic and orthotropic functionally graded materials (FGM) under mechanical and steady-state thermal loadings. Yu et al. [34] exploited XFEM for modeling the temperature field in heterogeneous materials, where the standard temperature field was enriched by using the level-set-based enrichment functions which model the interfaces. Macri et al. [35] presented a multiscale technique for modeling heterogeneous materials based on an enriched partition of unity that incorporates the thermal effects occurring on the microstructure into the global model for simulation. In Sapora et al. [36], an analogy between fracture and contact mechanics is proposed to investigate debonding phenomena at imperfect interfaces due to thermomechanical loading and thermal fields in bodies with cohesive cracks. From fracture mechanics point of view, Goli et al. [37] implemented the path-independent interaction integral in the context of the partition of unity for mixed mode adiabatic cracks under thermo-mechanical loadings particularly in orthotropic non-homogenous materials for a steady-state thermal problem. Bayesteh et al. [38] study a thermo-mechanical fracture of inhomogeneous cracked solids by the extended isogeometric analysis method, crack faces, and tip XFEM enrichment are incorporated into the non-uniform rational basis splines functions of isogeometric analysis (IGA) for static crack and steady-state thermal problem. Jia and Nie [39] adopted XFEM to analyze the interaction between a single or multiple macroscopic or microscopic inclusion and cracks for static crack and under the steady-state thermal problem. The work of Jaśkowiec [40] is concerned with modeling the heat flow through cracks in three-dimensional thermo-mechanical problems, the model for crack heat flow is combined with cohesive crack model. He et al. [41] established an XFEM thermo-elastic fracture problem for aluminium alloy metal inert gas welding, which includes a variable heat source with the initial and boundary conditions for a cracked plate structure. Li and Fish [42] developed a thermo-mechanical extended layerwise method for the laminated composite plates with delaminations and transverse cracks; transverse cracks are modeled using classical XFEM under pure mode-I. Recently, Zarmehri et al. [43] implemented XFEM to extract stress intensity factors for a stationary crack in an isotropic 2D finite domain under thermal shock, the coupled generalized thermo-elasticity theory employed is based on Green-Lindsay model.

Although the plethora of works has treated numerical thermo-mechanical analysis using classical XFEM recently, few works have employed the enhanced version of XFEM named XFEM-f.a. in order to ensure an optimal convergence through a geometrical enrichment regardless of the mesh size. This work aims at developing the complete full thermo-mechanical coupling using XFEM in adiabatic cracked media adopting a geometrical enrichment. The implementation was firstly validated for a single crack from the existing examples in the literature. Then, validation of the case of the combination between a hole and cracks, and the influence of crack size and a single hole size on the stress intensity factors, i.e., on the behavior of the rupture in a given structure, is performed. This case was investigated with the work of Prasad et al. based on the dual boundary element method for thermo-elastic crack problems [44]. Notably, the case of transient thermal loading and its impact on the SIFs profiles was treated, then a situation of the growth in mode-I was analyzed. Moreover, this work study the case of the thermo-mechanical propagation of multiple cracks in the presence of multiple holes in mixed mode.

This paper consists of six sections. The second one sketch the mathematical, physical and variational framework of the two-dimensional plane strain thermo-mechanical problem studied in a cracked medium. The third section intended for approximation spaces and the XFEM discretized forms of displacement and temperature fields as well as the full coupling XFEM matrices for each sub-problem part and the integration technique employed. Section four deals with the crack growth criterion assumed in this study, the form of the thermo-mechanical J-integral and the extraction of SIFs. Section five describes the specific numerical approach of a modified XFEM version involved. Several validation models from the literature are then considered for validation purpose; another benchmark with cracks and a manufacturing flaw idealized by a hole; an example of crack growth in mode-I, then in transient thermal loading; and lastly a mixed-mode crack growth model designed carefully for a specimen with multiple holes and cracks in the thermo-mechanical case. Finally, we conclude by a summary and some proposed extensions of this work.

## Problem and variational formulations

### Governing equations

**Fig. 1:**
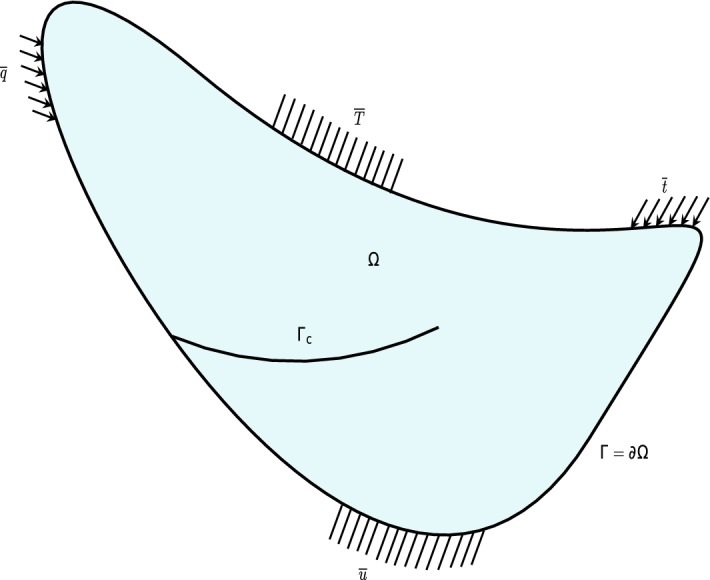
Cracked domain in two dimensions

Consider a linear-elastic, isotropic and homogeneous body occupying a geometrical cracked domain $\Omega$ bounded by $\Gamma =\partial \Omega$ in Fig. 1. The boundary $\Gamma$ is composed of parts $\Gamma _{u}$, $\Gamma _{T}$, $\Gamma _{t}$, $\Gamma _{q}$ and $\Gamma _{c}$. The equations of thermo-mechanical problem, assuming small displacements and small strains on $\Omega \setminus \Gamma _{c}$ are

1 $$\begin{aligned}&\rho c \displaystyle \frac{\partial T}{\partial t}(x,t) + \nabla \cdot q(x,t)\;=\;\overline{Q}(x,t) \end{aligned}$$

2 $$\begin{aligned}&q(x,t)\;=\;-k \nabla T(x,t) \end{aligned}$$

3 $$\begin{aligned}&\nabla \cdot \sigma (x,t) + \overline{b}(x) = 0 \end{aligned}$$

4 $$\begin{aligned}&\sigma (x,t) = \mathbb {C}:(\varepsilon -\varepsilon _{T})(x,t) \end{aligned}$$

5 $$\begin{aligned}&\varepsilon _{T}(x,t) \;=\;\alpha (T(x,t)-T_{0}(x))I \end{aligned}$$

6 $$\begin{aligned}&\varepsilon (x,t) \;=\;\nabla _{s}u(x,t) \end{aligned}$$

The objective is to find *u*(*x*, *t*) kinematically admissible, *T*(*x*, *t*), $\sigma (x,t)$ and *q*(*x*, *t*) for any $(x,t)\in (\Omega \setminus \Gamma _{c})\times ]0,\overline{T}_{f}]$, where, $\overline{T}_{f}$ is the end time. The fields are displacement vector *u*, temperature *T*, stress tensor $\sigma$, strain tensor $\varepsilon$, ’thermal strain’ tensor $\varepsilon _{T}$ defined with respect to a reference temperature $T_{0}$, heat flux vector *q*; the properties of materials are scalar thermal conductivity *k*, thermal expansion coefficient $\alpha$, density $\rho$, specific heat capacity *c*, and the isotropic fourth-order Hooke tensor $\mathbb {C}$, $\overline{Q}(x)$ and $\overline{b}(x)$ are respectively the imposed heat source and the body force on $\Omega \setminus \Gamma _{c}$. *I* is the second-order identity tensor and $\nabla _{s}$ is the symmetric gradient operator on a vector field. Prescribed displacements $\overline{u}$ and temperatures $\overline{T}$ are imposed respictively on $\Gamma _{u}$ and $\Gamma _{T}$, while tractions $t_{0}$ and heat flux $\overline{q}$ are imposed on $\Gamma _{t}$ and $\Gamma _{q}$ as

7 $$\begin{aligned}&u=\overline{u}\;\;\text{ on }\;\Gamma _{u},\;\;\sigma \cdot \;n=\overline{t}\;\;\text{ on }\;\Gamma _{t}, \;\;\sigma \cdot \;n=0\;\;\text{ on }\;\Gamma _{c}, \end{aligned}$$

8 $$\begin{aligned}&T=\overline{T}\;\;\text{ on }\;\Gamma _{T},\;\;q\cdot n=\overline{q}\;\;\text{ on }\;\Gamma _{q},\;\;q \cdot \;n=0\;\;\text{ on }\;\Gamma _{c}, \end{aligned}$$

The crack surface $\Gamma _{c}$ is assumed to be traction-free. The problem is well-defined with $\Gamma _{T}\cup \Gamma _{q}\subset \Gamma$, $\Gamma _{T}\cap \Gamma _{q}=\emptyset$ and $\Gamma _{u}\cup \Gamma _{t}\subset \Gamma$, $\Gamma _{u}\cap \Gamma _{t}=\emptyset$.

Thermal problem is merely time-dependent, while the mechanical one is quasi-static by neglecting inertial effects, Khoei et al. [45]. The time appeared in $\sigma (.,t)$, $\varepsilon (.,t)$ is a pseudo-time induced by the real-time in the time-space $]0,\overline{T}_{f}]$. In the staggered thermo-mechanical problem, which is not the case for this present work, the transient thermal problem is solved first to compute the temperature field at the real-time *t*, then the quasi-static mechanical problem defined by the equation Eq. (3) is solved by taking *T*(*t*) as input. Consequently, the resolution of the mechanical problem, in pseudo-time ’marching’, becomes conditioned *continuously* in real-time by the resolution of the thermal problem. Hence, by splitting the mechanical stress $\sigma (.,t)$ to a global stress $\sigma ^{\mathrm{g}}(.,t)$ and ’thermal stress’ $\sigma ^{\mathrm{th}\mathrm{th}}(.,t)$, the continuous space of mechanical pseudo-times can be defined for a traction-free crack by

9 $$\begin{aligned} \mathcal {PT}_{t}=\left\{ \tilde{t}:\nabla \cdot \sigma ^{\mathrm{g}}(x,\tilde{t})+\overline{b}=F(t); u|_{\Gamma _{u}}=\overline{u};\;\sigma \cdot n|_{\Gamma _{t}}=\overline{t}\;\;\text{ such }\;\;F(t)=\nabla \cdot \sigma ^{\mathrm{th}\mathrm{th}}(x,t)\right\} \end{aligned}$$

For a given real-time *t*, space $\mathcal {PT}_{t}$ will be reduced to a singleton. This definition explains that for each real-time a unique pseudo-time is defined implicitly and naturally with the temporal evolution of the system. This allows to simply consider $\tilde{t}\equiv t$. Also, from the numerical discretized point of view, it is possible to study a direct steady problem and to avoid the pseudo-time stepping with an associated small-time step in the non-steady state. Since, in this study, the problem is strongly coupled, Eqs. (1)–(6), the overall dynamic of the system is driven by the transient problem. Thus, the causal real-time retrace the ’dynamic’ of the mechanical problem which becomes pseudo-time-dependent. Subsequently, the entire problem is treated as a *monolithic* object, and all sub-problem parts progressed simultaneously.

Note that in case of several cracks, the mother crack $\Gamma _{c}$ can be decomposed into many *n* adiabatic cracks, $\Gamma _{c}=\bigcup ^{n}_{1}\Gamma _{c_{i}}$ such every $i \mathrm{th}\mathrm{th}$ crack remains adiabatic $q\cdot \;n=0$ and traction-free $\sigma \cdot \;n=0$ on each $\Gamma _{c_{i}}$, for any $i\in \llbracket 1,n\rrbracket$. Henceforth, we will present the XFEM developments for the mother crack, which remains valid for all sub-cracks.

### Variational form

The space of admissible displacement and temperature is $\mathcal {U}\times \mathcal {T}$, $X=(u,T)\in \mathcal {U}\times \mathcal {T}$, where variational spaces $\mathcal {U}$ and $\mathcal {T}$ are defined on Sobolev space $H^{1}(\Omega )$ by

$$\begin{aligned}&\mathcal {U}\;=\;\{u\in H^{1}(\Omega )^{2}\;:\;u=\overline{u}\;\;\text{ on }\;\;\Gamma _{u}\;\;\text{ and }\;\;u\;\;\text{ is } \text{ discontinuous } \text{ on }\;\;\Gamma _{c}\},\\&\mathcal {T}\;=\;\{T \in H^{1}(\Omega )\;:\;T=\overline{T}\;\;\text{ on }\;\;\Gamma _{T}\;\;\text{ and }\;\;T\;\;\text{ is } \text{ discontinuous } \text{ on }\;\;\Gamma _{c}\}, \end{aligned}$$

and the spaces of homogeneous essential conditions are given by

10 $$\begin{aligned}&\mathcal {U}_{0}\;=\;\{u\in H^{1}(\Omega )^{2}\;:\;u=0\;\;\text{ on }\;\;\Gamma _{u}\}\subset H^{1}_{0}(\Omega )^{2}, \end{aligned}$$

11 $$\begin{aligned}&\mathcal {T}_{0}\;=\;\{T \in H^{1}(\Omega )\;:\;T=0\;\;\text{ on }\;\;\Gamma _{T}\}\subset H^{1}_{0}(\Omega ) \end{aligned}$$

The weak form of the thermo-mechanical problem, with the test functions *v* and *w*, can be expressed as follow: Find $u\in \mathcal {U}$ and $T\in \mathcal {T}$ such

12 $$\begin{aligned} W^{u}_{t}(u,v)= & {} \int _{\Omega }\varepsilon (v):C:\varepsilon (u)d\Omega \;-\;\int _{\Omega }v\cdot \overline{b}\;d\Omega \;-\;\int _{\Gamma _{t}}v\cdot \overline{t}\;d\Gamma \;\nonumber \\&-\;\int _{\Omega }\varepsilon (v):C:\varepsilon _{T}(T)d\Omega \;=\;0,\;\;\forall v\in \mathcal {U}_{0} \end{aligned}$$

13 $$\begin{aligned} W^{T}(T,w;t)= & {} \int _{\Omega }w\left( \rho c \frac{\partial T}{\partial t}\right) d\Omega \;+\;\int _{\Omega }\nabla w\cdot \left( k\nabla T\right) d\Omega \;-\;\int _{\Omega }w\cdot \overline{Q}\;d\Omega \nonumber \\ \;&+\;\int _{\Gamma _{q}}w\cdot \overline{q}\;d\Gamma \;=\;0,\;\;\forall w\in \mathcal {T}_{0} \end{aligned}$$

## XFEM approximation and numerical integration

### Full coupled eXtended Finite Element form

Considering a finite element mesh $\mathcal {M}$ without taking account of the crack which is treated separately by an implicit description using the level-set method. The XFEM shifted discrete form of each component, $u\in \{u,v\}$, of the displacement field on $\mathcal {M}$ takes the following form:

14 $$\begin{aligned} u^{h}(x,y)\;= & {} \;\sum _{i\in N_{\mathcal {A}}}N_{i}(x,y)\mathbf u _{i}\;+\;\sum _{j\in N_{\mathcal {A}_{cr}}}\underbrace{N_{j}(x,y)[H(x,y)-H(x_{j},y_{j})]}_{\Phi _{j}}{} \mathbf b _{j}^{u}\nonumber \\&+ \sum _{k\in N_{\mathcal {A}_{tip}}}\underbrace{N_{k}(x,y)\sum _{l\in \llbracket 1,L\rrbracket } [F_{l}(r,\theta )-F_{l}(r_{k},\theta _{k})]}_{\Lambda _{k}}{} \mathbf c _{kl}^{u}, \end{aligned}$$

where $\mathcal {A}$ represents the whole set of nodes forming the mesh including all enriched nodes, $\mathcal {A}\subseteqq \mathcal {M}$; $\mathcal {A}_{cr}$ describes all the nodes building the elements crossed by the crack without tips, $\mathcal {A}_{cr}\subset \mathcal {A}$; and $\mathcal {A}_{tip}$ denotes the nodes constructing the tip elements, $\mathcal {A}_{tip}\subset \mathcal {A}$. $N_{\mathcal {A}}$, $N_{\mathcal {A}_{cr}}$ and $N_{\mathcal {A}_{tip}}$ are the countable sets of the nodes, respectively, of $\mathcal {A}$, $\mathcal {A}_{cr}$ and $\mathcal {A}_{tip}$. The singular asymptotic basis functions are given in polar coordinates $(r,\theta )$ by

15 $$\begin{aligned} \{F_{l}(r,\theta )\}=\sqrt{r}\left\{ \sin \frac{\theta }{2},\;\cos \frac{\theta }{2}, \;\sin \frac{\theta }{2}\cos \theta ,\;\cos \frac{\theta }{2}\cos \theta \right\} \end{aligned}$$

Similarly, the discrete form of the temperature field with a single asymptotic function, and with the same definitions mentioned above can be written as

16 $$\begin{aligned} T^{h}(x,y)\;= & {} \;\sum _{i\in N_{\mathcal {A}}}N_{i}(x,y)\mathbf T _{i}\;+\;\sum _{j\in N_{\mathcal {A}_{cr}}}\underbrace{N_{j}(x,y)[H(x,y)-H(x_{j},y_{j})]}_{\Phi _{j}}{} \mathbf b _{j}^{T}\nonumber \\&+ \sum _{k\in N_{\mathcal {A}_{tip}}}\underbrace{N_{k}(x,y)[F_{1}(r,\theta )-F_{1}(r_{k},\theta _{k})]}_{\Upsilon _{k}}{} \mathbf c _{k1}^{T}, \end{aligned}$$

namely that the asymptotic expression of the temperature field of an adiabatic crack can be expressed on the point $(r,\theta )$ in the polar reference centered on the corresponding crack-tip, Duflot [27], as

17 $$\begin{aligned} T = -\frac{K_{T}}{k}\sqrt{\frac{2r}{\pi }}\sin \left( \frac{\theta }{2}\right) \end{aligned}$$

Henceforth, we adopt the notations of $u:=u^{h}$ and $T:=T^{h}$ for numerical development, without making a difference between the continuous and the discrete form of both displacement and temperature.

Time discretization can be obtained by assuming that two displacement-temperature $\{X_{i}\}$ at time $t_{i}$ and $\{X_{i+1}\}$ at time $t_{i+1}$, $t_{i+1}=t_{i}+\Delta t$, are related by the generalized trapezoid rule, including a parameter to set $\beta$,

18 $$\begin{aligned} \{X_{i+1}\}\;=\;\{X_{i}\}+\left[ (1-\beta )\{\dot{X}_{i}\}+\beta \{\dot{X}_{i+1}\}\right] \Delta t \end{aligned}$$

For a given time-dependant linear system $[C]\{\dot{X}\}+[K]\{X\}=\{F\}$, we can write Eq. (18) for $t_{i}$ then for $t_{i+1}$, multiplying the first by $(1-\beta )$ and the second by $\beta$, adding the two resulting equations and eliminating the time derivative term for $t_{i+1}$ by Eq. (18); then, after some handling, we obtain the time-dependent scheme to compute $\{X_{i+1}\}$ at the actual time by

$$\begin{aligned} \left\{ \begin{array}{lll} \left( \beta [K]+\frac{1}{\Delta t}[C] \right) \{X_{i+1}\} = \beta \{F_{i+1}\}+[C]\left( \frac{1}{\Delta t}\{X_{i}\}+(1-\beta )\{\dot{X}_{i}\}\right) ,\\ \{\dot{X}_{i}\}= [C]^{-1}\left( \{F_{i}\}-[K]\{X_{i}\}\right) ,\\ \{X_{0}\}= X_{0}\\ \end{array} \right. \end{aligned}$$

To improve stability of the previous scheme, we choose the particular case of Crank–Nicolson, with $\beta =\frac{1}{2}$ which is unconditionally stable and with no numeric dissipation into the numerical approximation.

Let *X* be the global variable of the full XFEM thermo-mechanical coupling, such $\{X\}^{T}=\left\{ \{U_{\mathrm{std}}\}^{T},\{U_{\mathrm{enr}}\}^{T},\{T_{\mathrm{std}}\}^{T},\{T_{\mathrm{enr}}\}^{T}\right\}$; where $\{U_{\mathrm{std}}\}$ and $\{U_{\mathrm{enr}}\}$ are respectively the standard part and the enriched part of the displacement; the same goes for the temperature. The full coupling form of the XFEM stiffness matrix, damping matrix and force vector are given by19
20where $[\mathbf K _{UU}]$ is the purely mechanical part; $[\mathbf K _{TT}]$ is the purely thermal part; $[\mathbf K _{UT}]$ is the coupling part describes the influence of the thermal problem on the mechanical one; and the zero term matrix explains that there is no influence of the mechanical problem on the thermal one.

$$\bullet $$ Mechanical part:

The unknowns for mechanical problem are $\{U_{i},b_{i}^{u},c_{i}^{u}\}^{T}$; the mechanical stiffness matrix can be written as

21 $$\begin{aligned}{}[\mathbf K _{UU}]\;=\;\left[ \begin{array}{l@{\quad }l@{\quad }l@{\quad }l@{\quad }l@{\quad }l} [K_{uu}^{uu}] &{} [K_{uv}^{uu}] &{} [K_{uu}^{ub^{u}}] &{} [K_{uv}^{ub^{u}}] &{} [K_{uu}^{uc^{u}}] &{} [K_{uv}^{uc^{u}}]\\ &{} [K_{vv}^{uu}] &{} [K_{vu}^{ub^{u}}] &{} [K_{vv}^{ub^{u}}] &{} [K_{vu}^{uc^{u}}] &{} [K_{vv}^{uc^{u}}]\\ &{} &{} [K_{uu}^{bb^{u}}] &{} [K_{uv}^{bb^{u}}] &{} [K_{uu}^{bc^{u}}] &{} [K_{uv}^{bc^{u}}]\\ &{} &{} &{} [K_{vv}^{bb^{u}}] &{} [K_{vu}^{bc^{u}}] &{} [K_{vv}^{bc^{u}}]\\ &{} \text{ Sym. } &{} &{} &{} [K_{uu}^{cc^{u}}] &{} [K_{uv}^{cc^{u}}]\\ &{} &{} &{} &{} &{} [K_{vv}^{cc^{u}}] \end{array}\right] , \end{aligned}$$

where the expression of each sub-matrix component, with respect to the Lamé’s constants $\mu$ and $\lambda$ denoting in the indicial notation of the stress $\sigma _{ij}=2\mu \varepsilon _{ij}+[\lambda \varepsilon _{kk}-\alpha (3\lambda +2\mu )(T-T_{0})]\delta _{ij}$, can be given explicitly by

22 $$\begin{aligned}{}[K_{uu}^{uu}]\;= & {} \;\int _{\Omega }\left\{ (2\mu +\lambda )\frac{\partial N_{i}}{\partial x} \frac{\partial N_{j}}{\partial x} + \mu \frac{\partial N_{i}}{\partial y} \frac{\partial N_{j}}{\partial y}\right\} d\Omega , \end{aligned}$$

23 $$\begin{aligned} {[}K_{uv}^{uu}]\;= & {} \;\int _{\Omega }\left\{ \lambda \frac{\partial N_{i}}{\partial x} \frac{\partial N_{j}}{\partial y} + \mu \frac{\partial N_{i}}{\partial y} \frac{\partial N_{j}}{\partial x} \right\} d\Omega , \end{aligned}$$

24 $$\begin{aligned} {[}K_{vv}^{uu}]\;= & {} \;\int _{\Omega }\left\{ (2\mu +\lambda )\frac{\partial N_{i}}{\partial y} \frac{\partial N_{j}}{\partial y} + \mu \frac{\partial N_{i}}{\partial x} \frac{\partial N_{j}}{\partial x} \right\} d\Omega , \end{aligned}$$

and,

25 $$\begin{aligned}{}[K_{uu}^{ub^{u}}]\;= & {} \;\int _{\Omega }\left\{ (2\mu +\lambda )\frac{\partial N_{i}}{\partial x} \frac{\partial \Phi _{j}}{\partial x} + \mu \frac{\partial N_{i}}{\partial y} \frac{\partial \Phi _{j}}{\partial y}\right\} d\Omega , \end{aligned}$$

26 $$\begin{aligned} {[}K_{uv}^{ub^{u}}]\;= & {} \;\int _{\Omega }\left\{ \lambda \frac{\partial N_{i}}{\partial x} \frac{\partial \Phi _{j}}{\partial y} + \mu \frac{\partial N_{i}}{\partial y} \frac{\partial \Phi _{j}}{\partial x}\right\} d\Omega , \end{aligned}$$

27 $$\begin{aligned} {[}K_{vu}^{ub^{u}}]\;= & {} \;\int _{\Omega }\left\{ \lambda \frac{\partial N_{i}}{\partial y} \frac{\partial \Phi _{j}}{\partial x} + \mu \frac{\partial N_{i}}{\partial x} \frac{\partial \Phi _{j}}{\partial y}\right\} d\Omega , \end{aligned}$$

28 $$\begin{aligned} {[}K_{vv}^{ub^{u}}]\;= & {} \;\int _{\Omega }\left\{ (2\mu +\lambda )\frac{\partial N_{i}}{\partial y} \frac{\partial \Phi _{j}}{\partial y} + \mu \frac{\partial N_{i}}{\partial x} \frac{\partial \Phi _{j}}{\partial x} \right\} d\Omega , \end{aligned}$$

and,

29 $$\begin{aligned}{}[K_{uu}^{uc^{u}}]\;= & {} \;\int _{\Omega }\left\{ (2\mu +\lambda )\frac{\partial N_{i}}{\partial x} \frac{\partial \Lambda _{k}}{\partial x} + \mu \frac{\partial N_{i}}{\partial y} \frac{\partial \Lambda _{k}}{\partial y}\right\} d\Omega , \end{aligned}$$

30 $$\begin{aligned} {[}K_{uv}^{uc^{u}}]\;= & {} \;\int _{\Omega }\left\{ \lambda \frac{\partial N_{i}}{\partial x} \frac{\partial \Lambda _{k}}{\partial y} + \mu \frac{\partial N_{i}}{\partial y} \frac{\partial \Lambda _{k}}{\partial x}\right\} d\Omega , \end{aligned}$$

31 $$\begin{aligned} {[}K_{vu}^{uc^{u}}]\;= & {} \;\int _{\Omega }\left\{ \lambda \frac{\partial N_{i}}{\partial y} \frac{\partial \Lambda _{k}}{\partial x} + \mu \frac{\partial N_{i}}{\partial x} \frac{\partial \Lambda _{k}}{\partial y}\right\} d\Omega , \end{aligned}$$

32 $$\begin{aligned} {[}K_{vv}^{uc^{u}}]\;= & {} \;\int _{\Omega }\left\{ (2\mu +\lambda )\frac{\partial N_{i}}{\partial y} \frac{\partial \Lambda _{k}}{\partial y} + \mu \frac{\partial N_{i}}{\partial x} \frac{\partial \Lambda _{k}}{\partial x} \right\} d\Omega , \end{aligned}$$

and,

33 $$\begin{aligned}{}[K_{uu}^{bb^{u}}]\;= & {} \;\int _{\Omega }\left\{ (2\mu +\lambda )\frac{\partial \Phi _{i}}{\partial x} \frac{\partial \Phi _{j}}{\partial x} + \mu \frac{\partial \Phi _{i}}{\partial y} \frac{\partial \Phi _{j}}{\partial y}\right\} d\Omega , \end{aligned}$$

34 $$\begin{aligned} {[}K_{uv}^{bb^{u}}]\;= & {} \;\int _{\Omega }\left\{ \lambda \frac{\partial \Phi _{i}}{\partial x} \frac{\partial \Phi _{j}}{\partial y} + \mu \frac{\partial \Phi _{i}}{\partial y} \frac{\partial \Phi _{j}}{\partial x}\right\} d\Omega , \end{aligned}$$

35 $$\begin{aligned} {[}K_{vv}^{bb^{u}}]\;= & {} \;\int _{\Omega }\left\{ (2\mu +\lambda )\frac{\partial \Phi _{i}}{\partial y} \frac{\partial \Phi _{j}}{\partial y} + \mu \frac{\partial \Phi _{i}}{\partial x} \frac{\partial \Phi _{j}}{\partial x} \right\} d\Omega , \end{aligned}$$

and,

36 $$\begin{aligned}{}[K_{uu}^{bc^{u}}]\;= & {} \;\int _{\Omega }\left\{ (2\mu +\lambda )\frac{\partial \Phi _{i}}{\partial x} \frac{\partial \Lambda _{k}}{\partial x} + \mu \frac{\partial \Phi _{i}}{\partial y} \frac{\partial \Lambda _{k}}{\partial y}\right\} d\Omega , \end{aligned}$$

37 $$\begin{aligned} {[}K_{uv}^{bc^{u}}]\;= & {} \;\int _{\Omega }\left\{ \lambda \frac{\partial \Phi _{i}}{\partial x} \frac{\partial \Lambda _{k}}{\partial y} + \mu \frac{\partial \Phi _{i}}{\partial y} \frac{\partial \Lambda _{k}}{\partial x}\right\} d\Omega , \end{aligned}$$

38 $$\begin{aligned} {[}K_{vu}^{bc^{u}}]\;= & {} \;\int _{\Omega }\left\{ \lambda \frac{\partial \Phi _{i}}{\partial y} \frac{\partial \Lambda _{k}}{\partial x} + \mu \frac{\partial \Phi _{i}}{\partial x} \frac{\partial \Lambda _{k}}{\partial y}\right\} d\Omega , \end{aligned}$$

39 $$\begin{aligned} {[}K_{vv}^{bc^{u}}]\;= & {} \;\int _{\Omega }\left\{ (2\mu +\lambda )\frac{\partial \Phi _{i}}{\partial y} \frac{\partial \Lambda _{k}}{\partial y} + \mu \frac{\partial \Phi _{i}}{\partial x} \frac{\partial \Lambda _{k}}{\partial x} \right\} d\Omega , \end{aligned}$$

and,

40 $$\begin{aligned}{}[K_{uu}^{cc^{u}}]\;= & {} \;\int _{\Omega }\left\{ (2\mu +\lambda )\frac{\partial \Lambda _{i}}{\partial x} \frac{\partial \Lambda _{k}}{\partial x} + \mu \frac{\partial \Lambda _{i}}{\partial y} \frac{\partial \Lambda _{k}}{\partial y}\right\} d\Omega , \end{aligned}$$

41 $$\begin{aligned} {[}K_{uv}^{cc^{u}}]\;= & {} \;\int _{\Omega }\left\{ \lambda \frac{\partial \Lambda _{i}}{\partial x} \frac{\partial \Lambda _{k}}{\partial y} + \mu \frac{\partial \Lambda _{i}}{\partial y} \frac{\partial \Lambda _{k}}{\partial x}\right\} d\Omega , \end{aligned}$$

42 $$\begin{aligned} {[}K_{vv}^{cc^{u}}]\;= & {} \;\int _{\Omega }\left\{ (2\mu +\lambda )\frac{\partial \Lambda _{i}}{\partial y} \frac{\partial \Lambda _{k}}{\partial y} + \mu \frac{\partial \Lambda _{i}}{\partial x} \frac{\partial \Lambda _{k}}{\partial x} \right\} d\Omega \end{aligned}$$

The mechanical part of the force vector,

43 $$\begin{aligned} \{F_{U}\}^{T}\;= & {} \;\left\{ \;\{F_{u}^{u}\}^{T}\;\{F_{v}^{u}\}^{T}\;\{F_{u}^{b^{u}}\}^{T}\;\{F_{v}^{b^{u}}\}^{T}\;\{F_{u}^{c^{u}}\}^{T}\;\{F_{v}^{c^{u}}\}^{T}\right\} \end{aligned}$$

44 $$\begin{aligned} \{F_{u}^{u}\}= & {} \int _{\Omega }N_{i}\;\overline{b}_{1}\;d\Omega +\int _{\Gamma _{t}}N_{i}\;\overline{t}_{1}\;d\Gamma -\int _{\Omega }\beta \;\frac{\partial N_{i}}{\partial x}\;T_{0}\;d\Omega ;\nonumber \\ \{F_{v}^{u}\}= & {} \int _{\Omega }N_{i}\;\overline{b}_{2}\;d\Omega +\int _{\Gamma _{t}}N_{i}\;\overline{t}_{2}\;d\Gamma -\int _{\Omega }\beta \;\frac{\partial N_{i}}{\partial y}\;T_{0}\;d\Omega , \end{aligned}$$

45 $$\begin{aligned} \{F_{u}^{b^{u}}\}= & {} \int _{\Omega }\Phi _{j}\;\overline{b}_{1}\;d\Omega +\int _{\Gamma _{t}}\Phi _{j}\;\overline{t}_{1}\;d\Gamma -\int _{\Omega }\beta \;\frac{\partial \Phi _{j}}{\partial x}\;T_{0}\;d\Omega ;\nonumber \\ \{F_{v}^{b^{u}}\}= & {} \int _{\Omega }\Phi _{j}\;\overline{b}_{2}\;d\Omega +\int _{\Gamma _{t}}\Phi _{j}\;\overline{t}_{2}\;d\Gamma -\int _{\Omega }\beta \;\frac{\partial \Phi _{j}}{\partial y}\;T_{0}\;d\Omega , \end{aligned}$$

46 $$\begin{aligned} \{F_{u}^{c^{u}}\}= & {} \int _{\Omega }\Lambda _{k}\;\overline{b}_{1}\;d\Omega +\int _{\Gamma _{t}}\Lambda _{k}\;\overline{t}_{1}\;d\Gamma -\int _{\Omega }\beta \;\frac{\partial \Lambda _{k}}{\partial x}\;T_{0}\;d\Omega ;\nonumber \\ \{F_{v}^{c^{u}}\}= & {} \int _{\Omega }\Lambda _{k}\;\overline{b}_{2}\;d\Omega +\int _{\Gamma _{t}}\Lambda _{k}\;\overline{t}_{2}\;d\Gamma -\int _{\Omega }\beta \;\frac{\partial \Lambda _{k}}{\partial y}\;T_{0}\;d\Omega \end{aligned}$$

$$\bullet $$ Thermal part:

The unknowns for thermal problem are $\{T_{i},b_{i}^{T},c_{i}^{T}\}^{T}$; the thermal stiffness matrix can be written as

47 $$\begin{aligned} {[}{} \mathbf K _{TT}]\;=\;\left[ \begin{array}{lll} [K^{TT}] &{} [K^{Tb^{T}}] &{} [K^{Tc^{T}}] \\ &{} [K^{b^{T}b^{T}}] &{} [K^{b^{T}c^{T}}] \\ \text{ Sym. } &{} &{} [K^{c^{T}c^{T}}] \\ \end{array}\right] , \end{aligned}$$

where the expression of each sub-matrix component can be given explicitly by

48 $$\begin{aligned} {[}K^{TT}]\;= & {} \;\int _{\Omega }k\left\{ \frac{\partial N_{i}}{\partial x} \frac{\partial N_{j}}{\partial x} + \frac{\partial N_{i}}{\partial y} \frac{\partial N_{j}}{\partial y}\right\} d\Omega , \end{aligned}$$

49 $$\begin{aligned} {[}K^{Tb^{T}}]\;= & {} \;\int _{\Omega }k\left\{ \frac{\partial N_{i}}{\partial x} \frac{\partial \Phi _{j}}{\partial x} + \frac{\partial N_{i}}{\partial y} \frac{\partial \Phi _{j}}{\partial y}\right\} d\Omega , \end{aligned}$$

50 $$\begin{aligned} {[}K^{b^{T}b^{T}}]\;= & {} \;\int _{\Omega }k\left\{ \frac{\partial \Phi _{i}}{\partial x} \frac{\partial \Phi _{j}}{\partial x} + \frac{\partial \Phi _{i}}{\partial y} \frac{\partial \Phi _{j}}{\partial y}\right\} d\Omega , \end{aligned}$$

and

51 $$\begin{aligned} {[}K^{Tc^{T}}]\;= & {} \;\int _{\Omega }k\left\{ \frac{\partial N_{i}}{\partial x} \frac{\partial \Upsilon _{k}}{\partial x} + \frac{\partial N_{i}}{\partial y} \frac{\partial \Upsilon _{k}}{\partial y}\right\} d\Omega , \end{aligned}$$

52 $$\begin{aligned} {[}K^{b^{T}c^{T}}]\;= & {} \;\int _{\Omega }k\left\{ \frac{\partial \Phi _{i}}{\partial x} \frac{\partial \Upsilon _{k}}{\partial x} + \frac{\partial \Phi _{i}}{\partial y} \frac{\partial \Upsilon _{k}}{\partial y}\right\} d\Omega , \end{aligned}$$

53 $$\begin{aligned} {[}K^{c^{T}c^{T}}]\;= & {} \;\int _{\Omega }k\left\{ \frac{\partial \Upsilon _{j}}{\partial x} \frac{\partial \Upsilon _{k}}{\partial x} + \frac{\partial \Upsilon _{j}}{\partial y} \frac{\partial \Upsilon _{k}}{\partial y}\right\} d\Omega , \end{aligned}$$

The thermal part of the global force vector,

54 $$\begin{aligned} \{F_{T}\}^{T}\;= & {} \;\left\{ \;\{F^{T}\}^{T}\;\{F^{b^{T}}\}^{T}\;\{F^{c^{T}}\}^{T}\right\} , \end{aligned}$$

55 $$\begin{aligned} \{F^{T}\}\;= & {} \;\displaystyle \int _{\Omega }N_{i}\overline{Q}\;d\Omega - \int _{\Gamma _{q}}N_{i}\overline{q}\;d\Gamma ;\;\;\;\{F^{b^{T}}\}\;=\;\int _{\Omega }\Phi _{i}\overline{Q}\;d\Omega - \int _{\Gamma _{q}}\Phi _{i}\overline{q}\;d\Gamma ,\nonumber \\ \{F^{c^{T}}\}\;= & {} \;\displaystyle \int _{\Omega }\Upsilon _{i}\overline{Q}\;d\Omega - \int _{\Gamma _{q}}\Upsilon _{i}\overline{q}\;d\Gamma \end{aligned}$$

$$\bullet $$ Coupled part:

The thermo-mechanical coupled part can be expressed as

56 $$\begin{aligned} {[}{} \mathbf K _{UT}]\;=\;\alpha \left[ \begin{array}{l@{\quad }l@{\quad }l} [K_{u.}^{Tu}] &{} [K_{u.}^{b^{T}u}] &{} [K_{u.}^{c^{T}u}] \\ {[K_{v.}^{Tu}]} &{} [K_{v.}^{b^{T}u}] &{} [K_{v.}^{c^{T}u}] \\ {[K_{u.}^{Tb^{u}}]} &{} [K_{u.}^{b^{T}b^{u}}] &{} [K_{u.}^{c^{T}b^{u}}] \\ {[ K_{v.}^{Tb^{u}}]} &{} [K_{v.}^{b^{T}b^{u}}] &{} [K_{v.}^{c^{T}b^{u}}] \\ {[K_{u.}^{Tc^{u}}]} &{} [K_{u.}^{b^{T}c^{u}}] &{} [K_{u.}^{c^{T}c^{u}}] \\ {[K_{v.}^{Tc^{u}}]} &{} [K_{v.}^{b^{T}c^{u}}] &{} [K_{v.}^{c^{T}c^{u}}] \end{array}\right] = 2\alpha (\mu +\lambda )\left[ \begin{array}{l@{\quad }l@{\quad }l} N_{i}\frac{\partial N_{j}}{\partial x} &{} \Phi _{i}\frac{\partial N_{j}}{\partial x} &{} \Upsilon _{i}\frac{\partial N_{j}}{\partial x} \\ N_{i}\frac{\partial N_{j}}{\partial y} &{} \Phi _{i}\frac{\partial N_{j}}{\partial y} &{} \Upsilon _{i}\frac{\partial N_{j}}{\partial y} \\ N_{i}\frac{\partial \Phi _{j}}{\partial x} &{} \Phi _{i}\frac{\partial \Phi _{j}}{\partial x} &{} \Upsilon _{i}\frac{\partial \Phi _{j}}{\partial x} \\ N_{i}\frac{\partial \Phi _{j}}{\partial y} &{} \Phi _{i}\frac{\partial \Phi _{j}}{\partial y} &{} \Upsilon _{i}\frac{\partial \Phi _{j}}{\partial y} \\ N_{i}\frac{\partial \Lambda _{j}}{\partial x} &{} \Phi _{i}\frac{\partial \Lambda _{j}}{\partial x} &{} \Upsilon _{i}\frac{\partial \Lambda _{j}}{\partial x} \\ N_{i}\frac{\partial \Lambda _{j}}{\partial y} &{} \Phi _{i}\frac{\partial \Lambda _{j}}{\partial y} &{} \Upsilon _{i}\frac{\partial \Lambda _{j}}{\partial y} \end{array}\right] \end{aligned}$$

And the damping non-zero matrix $[\mathbf C _{TT}]$ is given by

57 $$\begin{aligned} {[}{} \mathbf C _{TT}]\;=\;\left[ \begin{array}{lll} [C^{TT}] &{} [C^{Tb^{T}}] &{} [C^{Tc^{T}}] \\ &{} [C^{b^{T}b^{T}}] &{} [C^{b^{T}c^{T}}] \\ \text{ Sym. } &{} &{} [C^{c^{T}c^{T}}] \\ \end{array}\right] , \end{aligned}$$

where each component can be expresses by,

58 $$\begin{aligned} {[}C^{TT}]= & {} \int _{\Omega }\rho cN_{i}N_{j}\;d\Omega ;\quad [C^{Tb^{T}}] = \int _{\Omega }\rho cN_{i}\Phi _{j}\;d\Omega \end{aligned}$$

59 $$\begin{aligned} {[}C^{b^{T}b^{T}}]= & {} \int _{\Omega }\rho c\Phi _{i}\Phi _{j}\;d\Omega ;\quad [C^{Tc^{T}}] = \int _{\Omega }\rho cN_{i}\Lambda _{j}\;d\Omega \end{aligned}$$

60 $$\begin{aligned} {[}C^{b^{T}c^{T}}]= & {} \int _{\Omega }\rho c\Phi _{i}\Lambda _{j}\;d\Omega ;\quad [C^{c^{T}c^{T}}]=\int _{\Omega }\rho c\Lambda _{i}\Lambda _{j}\;d\Omega \end{aligned}$$

### Numerical integration

**Fig. 2:**
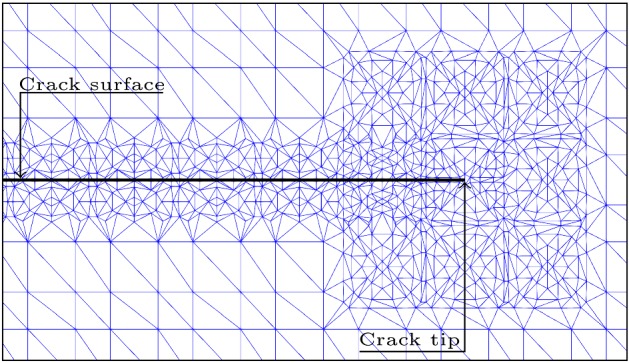
Integration procedure used: sub-triangulation mesh formed from Gauss points generated around surface and tip of the crack

In XFEM, the standard Gauss approximation approach cannot be used for the elements crossed by the crack. It is then necessary to modify it appropriately to evaluate the contribution of the weak form $W^{u}_{t}$ and $W^{T}$ for the two compartments generated by the crack at the sub-domain element $\Omega _{e}$ level. Indeed, the XFEM numerical integration requires a particular treatment due to the complexity encountered when integrating elements traversed by the discontinuity ($\Gamma _{c,e}=\Omega _{e}\cap \Gamma _{c}$) or crack-tip-element where the approximation functions are non-polynomials. The enrichment remains local in the vicinity of the crack region, termed *enriched-zone*; therefore, the area affected by this special treatment is located at the level of the enriched-zone. Beyond this zone, the elements are considered as standards with 4 or 8 integration points per non-enriched element. In the elements crossed completely by the crack $\Omega _{e}^{c}$, split or vertex element, the resulting configuration yields to a convex domain *C* and a complement non-convex domain $C^{c}_{\Omega _{e}^{c}}$. This situation needs a suitable geometrical approach, to deal with all possible cases, by a sub-polygons subdivision process to form a convex disjoint partition. A set of $m_{e}$ sub-convex-elements *K* of the same dimension taking account the relative position of the crack at the sub-domain element, such $\Omega _{e}=\bigcup _{1}^{m_{e}}K$, Dolbow et al. [19] and Laborde et al. [20], is taken. The same procedure can be done for tip-elements, or partially crossed by the crack, with much more attention due to the non-polynomial aspect of the functions of approximation. Commonly for both cases, the whole subdivision at the local element level can be seen as a *spider-web* delimited by the element borders, Fig. 2, centered on the crack-tip for the tip-element case and on the iso-barycenter of *C* or $C^{c}$ for split or vertex element cases.

In split element each sub-element take 7 integration points per *K*, in total we obtain $7*m_{e}$ points per split/vertex element. Also, we assume more integration points on the tip-elements to capture well numerically the singularity by 19 integration points per *K*; in total, we get $18*m_{e}$ per tip element. To note that to refine the XFEM approximation on the elements of transition between fully enriched elements and standard ones, we keep the same treatment as tip element with a spider-web centered on the iso-barycenter of the element.

### Enriched zone update in crack growth

In the initial state of propagation, the position of the crack is predefined $\Gamma _{c}^{0}$; the mesh $\mathcal {M}$ is properly generated, once and for all, without any change on it during the process of growth. Then, the relative position of the crack is implicitly identified by level-set functions, leaving aside its known Cartesian global position. Consequently, crack is recognized independently of the mesh definition, relatively, with respect to its nodal environment thanks to the signed-distance function. At this early stage, to write the discrete XFEM form of the displacement, Eq. (14), and the temperature, Eq. (16), fields, we ought to select the two kinds of enriched nodes families, Heaviside and tip enriched nodes. All the mesh nodes including those wholly enriched are roughly approximated by the standard shape functions. The nodes enriched by Heaviside function are described by the nodes forming the elements thoroughly crossed by the crack. The tip candidate nodes are such as those nodes composing the tip element for a topological enrichment; in geometrical enrichment on a given disk, $\mathcal {D}$ of radius *R* and centered on the tip, the discrete set of tip nodes is formed by the intersection of the mesh $\mathcal {M}$ and the disk $\mathcal {D}$, $\mathcal {M}\cap \mathcal {D}$. In geometrical enrichment instance, $\mathcal {D}$ crosses undoubtedly, for a large radius, the already selected Heaviside nodes. As a result, these nodes should be enriched simultaneously by the combined form of branching function and Heaviside, $F+H$. This selection configuration is performed for the initial increment and will identify the enriched-zone $EZ^{0}$ related to the position of the crack $\Gamma _{c}^{0}$ which is supposed to be unique. At the next increment, the same procedure is followed until a $(k\;-\;1)\mathrm{th}$ incremental crack is reached, $\Gamma _{c}^{k-1}$. At the $k\mathrm{th}$ increment, we suppose that the chosen crack growth criterion is satisfied, which allows extending the crack in the appropriate direction. This progress creates a new geometrical configuration of the crack and a new enriched-zone $EZ^{k}$ to be identified. We proceed in the same way, by recurrence, as the $(k\;-\;1)\mathrm{th}$ increment. This time we find ourselves with two different configurations where some nodes in the previous increment that they were, for example, of Heaviside type become tip nodes, or standard nodes convert to Heaviside/tip nodes, or vice versa. The transport of nodal fields corresponding to temperature and displacements computed at the $(k\;-\;1)\mathrm{th}$ increment to the $k\mathrm{th}$ one will be performed by an $L^{2}$-projection on the discrete space generated by the $k\mathrm{th}$ recent configuration by means of least squares method. This strategy is possible since the different quantities are square-integrable, which allows ensuring the stability and efficiency of the used scheme. To note that, the advancement of the crack generates a new geometrical, topological and numerical reality of the thermo-mechanical problem resolution which requires a specific treatment at each increment. This results in an extensible and flexible set of degrees of freedom with the crack evolution; therefore, the linear system of discrete equations is also extensible and changes in dimension depending on the crack growth state, it can enlarge or diminish. On the other hand, the selection rule of the $EZ^{k}$ nodes is independent of the previous configuration and related only to the relative position of the crack in its nodal environment at the actual increment. We are thus left with two different configurations of two successive increments. This process is repeated iteratively until the estimated or evaluated end of the propagation process.

## Crack growth criterion and stress intensity factors evaluation

### Propagation criterion and crack update

It has been shown that the use of level-set function plays an essential role in the implicit description of the crack and evaluation of enriched fields, mechanical Moës et al. [6] and thermal in this work. The crack is representing the zero-level set of a given function. The crack tip positions can be found by considering the intersection between zero-level contour and a second orthogonal level-set function Stolarska et al. [25] using the signed-distance function. The signed-distance in the level-set method is represented by a finite element approximation with the same mesh used for the mechanical and thermal problems. Adopting this representation makes the task easier when it is necessary to evaluate the level-set at element level by interpolation and when we need to compute its derivative which is well-defined by the derivative of shape functions.

To monitoring crack growth, we use the maximum hoop (circumferential) tensile stress theory introduced firstly by Erdogan and Sih [46]. In mixed-mode, the information is extracted in the vicinity of the crack tip by evaluating the stress state, written in polar coordinates. We assume that the crack extension starts at its tip in a radial direction, it is produced in the plane perpendicular to the direction of uttermost tension, i.e., at a critical angle $\theta _{c}$, and it begins when $\sigma _{\theta \theta }$ reaches a critical given value. When $K_{II}=0$ then $\theta _{c}=0$ also, in this case, we have a pure mode-I. By considering $K_{II}<0$ the critical crack growth $\theta _{c}>0$, and if $K_{II}>0$ the angle $\theta _{c}<0$. A handy expression of $\theta _{c}$ was given by Sukumar [7],

61 $$\begin{aligned} \theta _{c}=2\tan ^{-1}\left[ \frac{-2(K_{II}/K_{I})}{1+\sqrt{1+8(K_{II}/K_{I})^{2}}}\right] \end{aligned}$$

The extension of the crack path is determined by a constant increment of growth as an attractive approach. The selection of $\Delta a$ is almost always made *a priori* as an input parameter of the numerical crack propagation model. Several settings affect the quality of crack propagation path; those factors are widely studied by [8] using many examples illustrating the impact of these choices on the path. Therefore, it is more judicious numerically to choose a $\Delta a$ that takes into account those number of parameters Belytschko [2] to ensure convergence toward the appropriate path. Principally, three parameters influence the quality of the crack path: Firstly the $\textit{crack growth magnitude}$ (length of the crack incremental segment) which have to be considered within a range of $l_{e}\le \Delta a\le \frac{3}{2}l_{e}$, such, the element size $l_{e}=\sqrt{A_{e}}$ and $A_{e}$ is the average area of the elements. Secondly, the $\textit{mesh size}$ is important to have the best approximation of the field near crack with a finer one. Finally, the choice of $\textit{J-integral domain}$ is decisive to evaluate adequately the J-integral which allow extracting stress intensity factors in mode-I, II and in mixed mode and determining after that the value of crack growth orientation $\theta _{c}$.

On the other hand, different crack extension criteria exist in the literature and adequately ensures the crack progression, governed by fatigue law varieties. They are adapted to the crack progress when it is subjected to cyclic loading. The crack rate increment with respect to the loading cycle, i.e., speed growth, appeared in these laws and assumed to be, in general, a function which depends on the stress intensity factor range between two cycles and the stress ratio, Beden et al. [47]. The popular one is the classical law of Paris which is a version of the general law of fatigue, where the speed growth depends on the stress intensity factor range, and two constants, called constants of Paris law, that have to be identified for each specific material, Cherepanov et al. [48]. Its limitation lies in the fact that it requires a minimum stress intensity factor to ensure the propagation and does not take into account the stress ratio. Another version appeared later by Xiaoping et al. [49] that overcomes these limitations of the classical Paris law but requires three additional parameters more than the classical Paris law. All these models can ‘better’ capture the crack progress and monitor the history of the adapted crack increment for each promotion. They are more suited to fatigue propagation fashion and also require additional parameters related to the material that can be determined by fatigue tests. This last point may be a drawback for the attractiveness of these methods for the present work. However, the convergence of fixed crack increment method may be ‘lower’ in some cases, but with a suitable choice of the crack increment, which depends on the mesh and other parameters as cited previously, one can reach good results. Besides, fixed crack increment technique is more attractive; it needs less material parameters compared with the earlier mentioned laws. Its ability to obtain crack paths that coincide very well with reference solutions is investigated by Baydoun et al. [50].

### Stress intensity factors evaluation

**Fig. 3:**
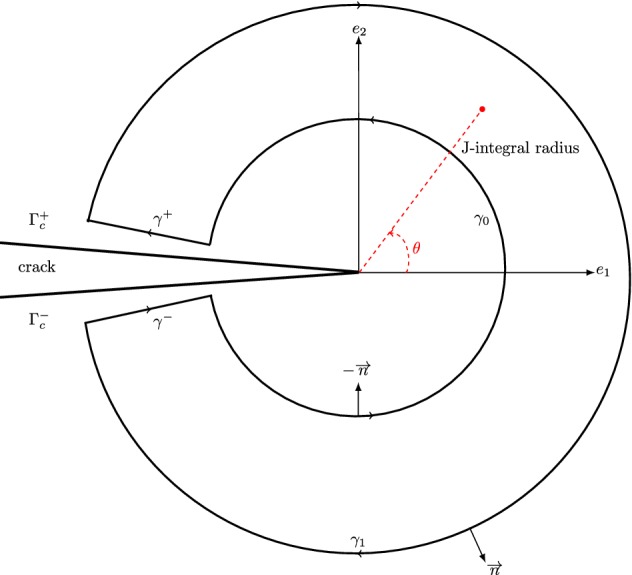
Arbitrary J-integral area surrounding the crack tip

The J-integral, with free body force $\overline{b}$, was introduced by Rice [51] as a way to compute the energy release rate *G*. Rice defined a line path independent integral, which keeps the same value for any path surrounding crack tip as

62 $$\begin{aligned} J=\lim _{\Gamma _{\varepsilon } \longrightarrow 0}\int _{\Gamma _{\varepsilon }}\left[ W\delta _{1i}-\sigma _{ij}\frac{\partial u_{j}}{\partial x_{1}}\right] n_{i}d\Gamma , \end{aligned}$$

where $\delta _{..}$ is the Kronecker operator, $n_{i}$ is the component in i-direction of the normal outward vector to the contour $\Gamma _{\varepsilon }$, $t_{j}=\sigma _{ij}n_{i}$ and $u_{j}$ are components of the interior traction and displacements, *W* is the strain energy density per unit volume defined in the thermo-mechanical state by

63 $$\begin{aligned} W\;=\;\frac{1}{2}\sigma _{ij}\varepsilon _{ij}^{m}=\frac{1}{2}\sigma _{ij}(\varepsilon _{ij}^{t}-\alpha \Delta T\delta _{ij}), \end{aligned}$$

where $\Delta T = T - T_{0}$, $\varepsilon _{ij}^{m}$ represents the mechanical part of strain and $\varepsilon _{ij}^{t}$ denotes the total strain. The form of the integral (62) is not adapted for a finite element computation, in particular in XFEM, while preserving the same shape functions. An enclosed contour $\Gamma ^{*}$ is considered as a sum of piecewise lines as defined in Fig. 3, $\Gamma ^{*}=\gamma ^{+}\cup \gamma _{0}\cup \gamma ^{-}\cup \gamma _{1}$. Hence, the J-integral can be converted into a domain integral by introducing a weight function *q* in the expression of (62), that is unity on $\gamma _{0}$, zero on $\gamma _{1}$ and varying monotonically in-between. In this work, we used a plateau truncated cone. By applying the divergence theorem, the equivalent domain integral (EDI) form of the J-integral is obtained as

64 $$\begin{aligned} J=\int _{A}(\sigma _{ij}u_{i,1}-W\delta _{1j})q_{,j}\;dA+\int _{A}(\sigma _{ij}u_{i,1}-W\delta _{1j})_{,j}q\;dA \end{aligned}$$

The J-integral of the superimposed of two equilibrium states: State 1 with *u*, $\sigma$ and $\varepsilon$ corresponds to the *real* state and state 2 with $u^{\mathrm{aux}}$, $\sigma ^{\mathrm{aux}}$ and $\varepsilon ^{\mathrm{aux}}$ corresponds to an *auxiliary* situation, is given by

65 $$\begin{aligned} J^{s}(\sigma +\sigma ^{\mathrm{aux}},\varepsilon +\varepsilon ^{\mathrm{aux}},u+u^{\mathrm{aux}})\;=\;J(\sigma ,\varepsilon ,u)+J(\sigma ^{\mathrm{aux}},\varepsilon ^{\mathrm{aux}},u^{\mathrm{aux}})+I, \end{aligned}$$

which can be explicitly written as

$$\begin{aligned} J^{s}= & {} \int _{A} \left\{ (\sigma _{ij}+\sigma _{ij}^{\mathrm{aux}}) (u_{i,1}+u_{i,1}^{\mathrm{aux}}) - \frac{1}{2}(\sigma _{ik}+\sigma _{ik}^{\mathrm{aux}}) (\varepsilon _{ik}^{m}+\varepsilon _{ik}^{\mathrm{aux}})\delta _{1j}\right\} q_{,j}\;dA\\&+ \int _{A} \left\{ (\sigma _{ij}+\sigma _{ij}^{\mathrm{aux}}) (u_{i,1}+u_{i,1}^{\mathrm{aux}}) - \frac{1}{2}(\sigma _{ik}+\sigma _{ik}^{\mathrm{aux}}) (\varepsilon _{ik}^{m}+\varepsilon _{ik}^{\mathrm{aux}})\delta _{1j}\right\} _{,j}q\;dA \end{aligned}$$

By developing $J^{s}$, the interaction integral *I* is obtained by

66 $$\begin{aligned} I\;= & {} \;\int _{A} \left\{ (\sigma _{ij}u_{i,1}^{\mathrm{aux}}+\sigma _{ij}^{\mathrm{aux}}u_{i,1})-\frac{1}{2}(\sigma _{ik}\varepsilon _{ik}^{\mathrm{aux}}+\varepsilon _{ik}^{\mathrm{aux}}\varepsilon _{ik}^{m})\delta _{1j}\right\} q_{,j}\;dA\nonumber \\&+ \int _{A} \left\{ (\sigma _{ij}u_{i,1}^{\mathrm{aux}}+\sigma _{ij}^{\mathrm{aux}}u_{i,1}^{\mathrm{aux}})-\frac{1}{2}(\sigma _{ik}\varepsilon _{ik}^{\mathrm{aux}}+\varepsilon _{ik}^{\mathrm{aux}}\varepsilon _{ik}^{m})\delta _{1j}\right\} _{,j}q\;dA \end{aligned}$$

By assuming crack faces to be traction free, using equilibrium (i.e., $\sigma _{ij,j}$=0), strain-displacement equations, and after some handling, we obtain

67 $$\begin{aligned} I\;= & {} \;\int _{A} \left\{ (\sigma _{ij}u_{i,1}^{\mathrm{aux}}+\sigma _{ij}^{\mathrm{aux}}u_{i,1})-\sigma _{ik}\varepsilon _{ik}^{\mathrm{aux}}\delta _{1j}\right\} q_{,j}\;dA\nonumber \\&+ \int _{A} \alpha \sigma _{ij}^{\mathrm{aux}}(\Delta T)_{,1}\delta _{ij}q\;dA \end{aligned}$$

For general mixed mode problems and isotropic materials, the direct relationship between J-integral and the stress intensity factors, having dimensions of [stress.$\sqrt{\text{ lenght }}$ ], in mode I and II is given by

68 $$\begin{aligned} J=\frac{K_{I}^{2}}{E^{*}}+\frac{K_{II}^{2}}{E^{*}}, \end{aligned}$$

where $E^{*}=E$ for plane stress and $E^{*}=E/(1-\nu ^2)$ for plane strain. Equations (65) and (68) leads to the following expression

69 $$\begin{aligned} I\;=\;\frac{2}{E^{*}}\left( K_{I}K_{I}^{\mathrm{aux}}+K_{II}K_{II}^{\mathrm{aux}}\right) \end{aligned}$$

The extraction of individual mode-I and mode-II stress intensity factors can be done by the choice of $K_{I}^{\mathrm{aux}}=1$ and $K_{II}^{\mathrm{aux}}=0$ to find $K_{I}$ and $K_{I}^{\mathrm{aux}}=0$ and $K_{II}^{\mathrm{aux}}=1$ to find $K_{II}$ as

70 $$\begin{aligned} K_{I}=\frac{E^{*}}{2}I^{(1)}\;\;\;\text{ and }\;\;\;K_{II}=\frac{E^{*}}{2}I^{(2)} \end{aligned}$$

The identification of SIFs and update of the crack by LSM after the computation of the thermal and mechanical responses by XFEM makes it possible to present now some examples of validation.

## Numerical examples of thermo-mechanical analysis

A set of thermo-mechanical examples are herein discussed by considering a strong material discontinuity; for a static adiabatic crack and in propagation state of an isotropic material. Validation of the results is fulfilled by a comparison with the computation of the stress intensity factors which allows validating both mechanical and thermal responses as well as the quantification of the linear elastic fracture mechanics (LEFM) parameters. The computation domains chosen for the benchmarks are extracted from the literature and meshes are generated using Gmsh [52]. A hybrid object-oriented code has been developed in a monolithic multi-physical philosophy treating each step starting from the mesh generated from Gmsh, the definition of the enrichment-zone, the XFEM matrix computation blocks associated to each physical segment and to each coupled part, the computation of fracture mechanics quantities and post-processing context.

The rate of convergence of conventional XFEM, using a ‘topological’ enrichment, is not improved when the characteristic mesh length *h* goes to zero because of the presence of a singularity. Laborde et al. [20] proposed a modified version of XFEM by enriching a whole fixed area (f.a) around the crack-tip, named XFEM-f.a. In the standard XFEM, only the nodes of the crack tip element are enriched by branching functions, the support of the additional basis functions vanishes when *h* is going to zero. In two dimensions, the fixed enriched area of a radius $E_{R}^{j}$ according to the $j\mathrm{th}$ crack-tip is giving by the disk

71 $$\begin{aligned} \mathcal {D}_{j}(E_{R}^{j}) = \left\{ x\in \Omega \setminus \Gamma _{c},\;\Vert x-x_{tip}^{j}\Vert \leqslant E_{R}^{j}\right\} \end{aligned}$$

The major drawback of ‘topological’ enrichment is that the size of the enriched zone depends linearly on the size of the mesh. However, ‘geometrical’ enrichment has an asset by enriching all the elements containing in the disk $\mathcal {D}_{.}$ for a given radius $E_{R}^{.}$ regardless of the mesh size. Therefore, XFEM-f.a. achieves the expected optimal rate of convergence of *O*(*h*). For a given configuration where several singularities (crack-tips) are apparently present, the fixed enriched area defined by gathering the multiple disks assigned to each singularity, ensuring that they remain disjointed by a judicious choice of the radius of each disk. Then, the global f.a. is given by

72 $$\begin{aligned} \overline{\mathcal {D}}=\left\{ \bigcup _{j\in N_{tip}}\mathcal {D}_{j}(E_{R}^{j})\;;\;\mathcal {D}_{j}(E_{R}^{j})\cap \mathcal {D}_{i}(E_{R}^{i}) = \emptyset \;\text{ for } \text{ each }\;j\ne i \right\} , \end{aligned}$$

where $N_{tip}$ is the discrete set of crack-tips. The discrete approximations of displacement, Eq. (14), and temperature, Eq. (16), by XFEM keep the same expression with a significant change in the topological enrichment of the crack-tip. Thus, the set $N_{\mathcal {A}_{tip}}$ of the nodes enriched by branching functions is transformed to $N_{\overline{\mathcal {D}}}$ which represents all the nodes forming the geometrical enrichment zone established by $\overline{\mathcal {D}}$. It is noteworthy that the effect of the blending elements decreases systematically with the increase of the enrichment area on the whole $\overline{\mathcal {D}}$. Also, there is no significant effect observed on the numerical solutions Fries [53].

Numerical results are performed for a full thermo-mechanical coupling problem in a cracked domain using a plane strain analysis, where the mechanical loading is induced by a pure thermal one under the prescribed temperatures and flux on boundaries. This case represents the most relevant situation, which can be easily combined with a pure mechanical load acted by external forces. One can simplify the analysis by considering $\Theta =T\;-\;T_{0}$, with $T_{0}=0$ initially for the whole domain, and with no heat source $\overline{Q}$ and no body force $\overline{b}$, which is the case for our analyzes. The crack surface is thermally insulated, so the flux lines have to circumvent the crack. Stress intensity factors computation is commonly normalized with respect to another choice of the triplet $(E, k, \alpha )$ material and with a fixed value of Poisson ration to 0.3. Normalized SIFs are presented for all the examples, including the negative values of $K_{I}$, for a static crack, which represents an important indicator of the compressive effect at the crack lips. The contact between crack surfaces is not taken into account in the numerical model, leaving XFEM-f.a. to produce information that can predict an inter-penetration of crack faces for certain thermo-mechanical configurations/domains. This plight remains entirely true, valid and adequate from a conceptual point of view. The J-integral radius is considered as a function of enrichment radius and have to be greater than or equal to $E_{R}^{j}$ to obtain good results of SIFs computations and taken in general for all cases, unless otherwise stated, equal to $\frac{3}{2}\times E_{R}^{j}$. Material parameters are set by default for all the examples, unless otherwise stated, by Table 1.

**Table 1:** Material properties

Poisson ratio-$\nu$	$0.3 \ [-]$
Young’s modulus-*E*	$2.184*10^{5}$ [Pa]
Thermal conductivity-*k*	205 [$\mathrm{W}\;\mathrm{m}^{-1}\;^\circ$C$^{-1}$]
Thermal expansion coefficient-$\alpha$	$1.67*10^{-5}$ [$^\circ$C$^{-1}$]

**Fig. 4:**
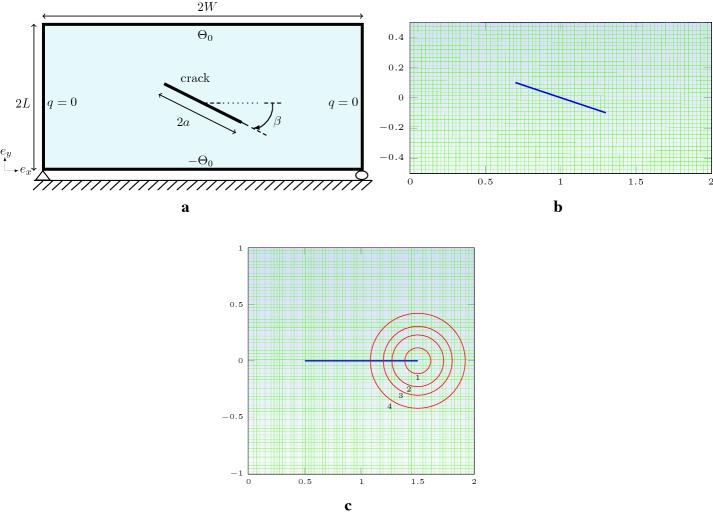
Rectangular plate with a slope crack: **a** thermo-mechanical boundary conditions, **b** structured mesh used for the computation, **c** J-integral paths used for a square plate with a centred crack

In this section, we present various examples to validate the thermo-mechanical model by XFEM-f.a. implementation, comparing with several benchmarks taken from the literature. The primary objective of all these cases is to investigate the accuracy and robustness of the numerical results. Then, we present an example of the cracked domain under transient-thermal load and in crack growth governed by mode-I. Finally, we design a model of crack propagation in mixed mode for a pure thermal loading, with round holes and multiple cracks.

### Rectangular plate with a centered slope crack

A rectangular plate specimen with a centered inclined crack subjected to a pure thermal load is analyzed, with the dimensions 2*L*, 2*W*, the crack is defined with the half-length *a* and the slope is characterized by the $\beta$ angle Fig. 4a. The displacements along the $e_{y}$-axis is fixed at the bottom extreme right corner, and the bottom left corner is clamped. Both right and left boards are completely insulated, an imposed temperatures of $\pm \Theta _{0}$ are defined at the top and bottom sides, such $\Theta _{0}=10\;^\circ$C. We consider a uniform enrichment disks radius in the case where several crack-tips exist; hence, $E_{R}\equiv E_{R}^{j}$. The radius of disks enrichment $E_{R}$ is taken equal to $0.15\;\mathrm{m}$ for both rectangular and square plates examples. We divide this example into two cases.

$\textit{First}$, a particular case of a square plate with a centered horizontal crack is considered, $L=W=2.0\;\mathrm{m}$ and $\beta =0^\circ$, Fig. 4c. The objective of this example is, firstly, to study the accuracy of J-integral computation independently of the choice of Rice integral contour. Secondly, to show the robustness of computation of stress intensity factors in the dominated mode-II for various horizontal crack lengths. The stress intensity factors are normalized by dividing $K_{II}$ by $\alpha \Theta _{0}E\sqrt{W}$ which gives $K_{II}^{\mathrm{norm}}$. In Table 2, the numerical normalized SIFs results are presented for four selected paths centered on right crack tip, referred by numbers ‘1’ to ‘4’. There is no difference related to the choice of the right or left tip. The physical domain is discretized with a structured quadrilateral mesh with a characteristic length of $0.016\;\mathrm{m}$. The variation of the SIFs values remains in the range $[0,0.9482\%]$ with a maximum variation of $0.94\%$ with respect to the minimum value, which corresponds to Path 2. The results obtained with XFEM-f.a. show a good outcome for path independence.

**Table 2:** Normalized SIFs, various J-integral paths

	**Paths**					**References**		
	**Path 1**	**Path 2**	**Path 3**	**Path 4**	**Average**	[54]	[44]	[27]
Radius [m]	0.1139	0.2278	0.3037	0.4176				
$K_{II}^{\mathrm{norm}}$	0.189978	0.189497	0.190830	0.191294	0.190399	0.188	0.190	0.191

**Table 3:** Normalized SIFs for centred crack in a square plate, various *a* / *W*

$\varvec{\frac{a}{W}}$	$\varvec{K}_{\varvec{II}}^{\mathrm{norm}}$			
	**Present work**	**Murakami** [54]	**Prasad et al.** [44]	**Duflot** [27]
0.1	0.0181	0.021	0.018	0.019
0.2	0.0535	0.053	0.054	0.054
0.3	0.0966	0.094	0.095	0.096
0.4	0.1412	0.141	0.141	0.141
0.5	0.1920	0.188	0.190	0.191
0.6	0.2480	0.247	0.243	0.245

**Fig. 5:**
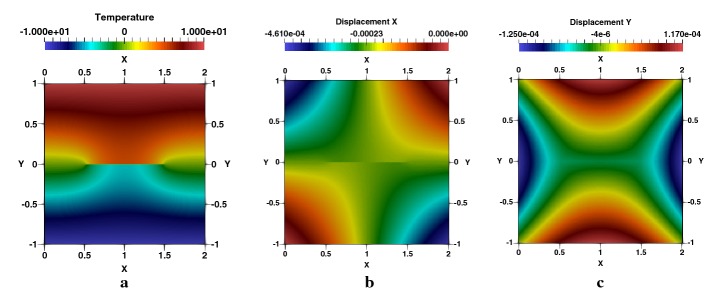
Square plate with a centred crack, $a/W=0.5$: **a** temperature, **b** $e_{x}$-displacement and **c** $e_{y}$-displacement

**Fig. 6:**
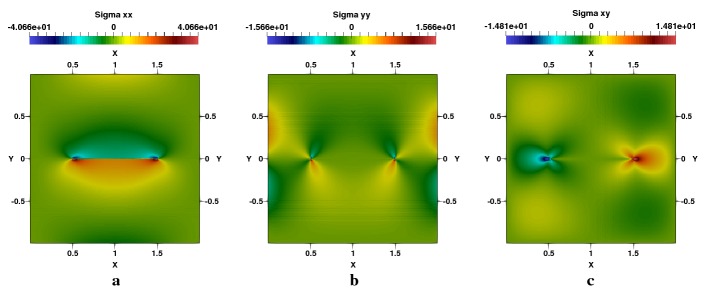
Square plate with a centred crack, $a/W=0.5$: **a** $\sigma _{xx}$-stress, **b** $\sigma _{yy}$-stress, **c** $\sigma _{xy}$-stress

**Fig. 7:**
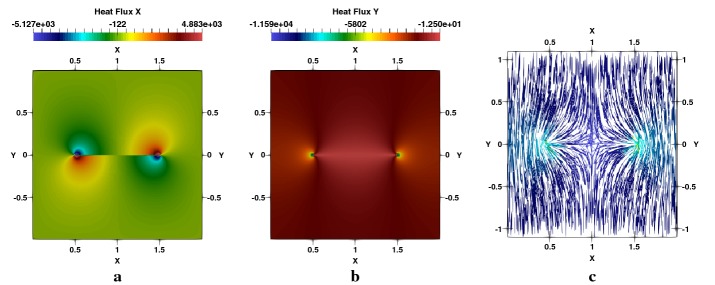
Square plate with a centred crack, $a/W=0.5$: **a** $q_{x}$-heat flux, **b** $q_{y}$-heat flux, **c** flux lines

Next, we consider different crack lengths starting from 0.1 to 0.6 with a jump of 0.1 with the same specimen configuration. The normalized SIFs results obtained with XFEM-f.a. agree closely with those presented by Murakami [54], Prasad et al. [44] and Duflot [27] for each crack length as shown shortly in Table 3 with respect to the results presented by the previous cited references. Complete results of this example are presented in Appendix: Table 7, with 6 digits, including the negative values of SIFs illustrating, as an indicator, of an important compressive aspect in the vicinity of the two tips. Temperature distribution is illustrated in Fig. 5a, as well as the $e_{x}$, in Fig. 5b, and $e_{y}$, in Fig. 5c, displacements. An important concentration of the stresses at the crack-tips are observed by Fig. 6a–c. Thermal flux is perpendicular to the crack Fig. 7a, b, since the crack is adiabatic; one notes a gradual deviation of the flux lines to circumvent the geometry of the crack Fig. 7c. These results are not presented by the references cited above.

**Table 4:** Normalized SIFs for slope crack, $\beta =30^\circ$ and various *a* / *W*

$\varvec{\frac{a}{W}}$	$\varvec{K}_{\varvec{I}}^{\mathrm{norm}}$				$\varvec{K}_{\varvec{II}}^{\mathrm{norm}}$			
	**Present work**	[54]	[44]	[27]	**Present work**	[54]	[44]	[27]
0.2	0.0021	0.002	0.002	0.0020	0.0301	0.030	0.030	0.0302
0.3	0.0069	0.008	0.006	0.0068	0.0484	0.048	0.048	0.0489
0.4	0.0152	0.015	0.014	0.0149	0.0640	0.064	0.064	0.0650
0.5	0.0269	0.027	0.026	0.0265	0.0773	0.076	0.076	0.0774
0.6	0.0408	0.041	0.040	0.0407	0.0872	0.086	0.087	0.0878

**Fig. 8:**
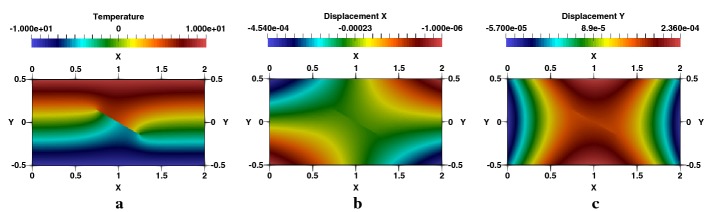
Rectangular plate with inclined crack, $\beta =30^\circ$ and $a/W=0.3$: **a** temperature, **b** $e_{x}$-displacement, **c** $e_{y}$-displacement

**Fig. 9:**
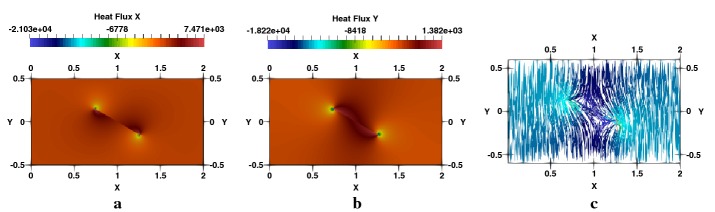
Rectangular plate with inclined crack, $\beta =30^\circ$ and $a/W=0.3$: **a** $q_{x}$-heat flux, **b** $q_{y}$-heat flux, **c** flux lines

**Fig. 10:**
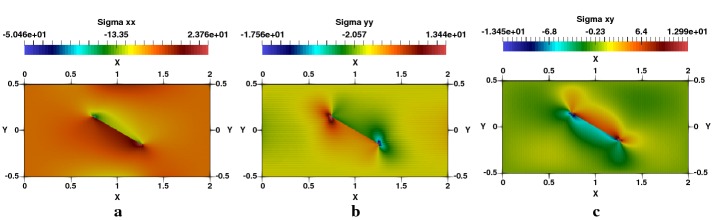
Rectangular plate with inclined crack, $\beta =30^\circ$ and $a/W=0.3$: **a** $\sigma _{xx}$-stress, **b** $\sigma _{yy}$-stress, **c** $\sigma _{xy}$-stress

$\textit{Second}$, as a benchmark problem, we treat the general case of any choice of $\beta \in [0,\frac{\pi }{2}]$ and various choices of crack lengths. Dimensions is chosen such $L/W=0.5$, inclined crack is defined by the total crack length 2*a* Fig. 4b. The main objective of this example is to show the accuracy and the robustness at the same time to predict mixed mode $K_{I}$ and $K_{II}$ stress intensity factors. The stress intensity factors are normalized by $\alpha \Theta _{0}(W/L) E\sqrt{2W}$ which gives $K_{I}^{\mathrm{norm}}$ and $K_{II}^{\mathrm{norm}}$ correspond respectively to mode-I and mode-II. Table 4 summarizes the results of normalized SIFs for a fixed angle $\beta =30^\circ$ and various crack lengths varying from 0.2 to 0.6. In Table 5, we give the normalized SIFs results for a fixed crack length, here $a/W=0.3$, and different values of $\beta$. Complete results of both cases are given respectively in Appendix: Tables 8 and 9 with 6 digits, including again the negative values of SIFs. Results are observed to be in good agreement with Murakami [54], Prasad et al. [44] and Duflot [27]. Temperature distribution influenced by the prescence of crack, $e_{x}$ and $e_{y}$ displacements are presented respectively in Fig. 8a–c. Horizontal, vertical and line flux are plotted respectively in Fig. 9a–c. Stresses, Fig. 10a–c, show the same behavior around the crack-tips like in the case of the square plate. Again, these results are not presented by the references cited above.

**Table 5:** Normalized SIFs for slope crack at both tips, $a/W=0.3$ and various $\beta$

$\varvec{\frac{a}{W}}$	$\varvec{K}_{\varvec{I}}^{\mathrm{norm}}$				$\varvec{K}_{\varvec{II}}^{\mathrm{norm}}$			
	**Present work**	[54]	[44]	[27]	**Present work**	[54]	[44]	[27]
$0^\circ$	0.0000	0.0000	0.0000	0.0000	0.0548	0.054	0.054	0.0546
$15^\circ$	0.0036	0.0038	0.0036	0.0038	0.0533	0.054	0.054	0.0533
$30^\circ$	0.0069	0.0071	0.0064	0.0068	0.0484	0.048	0.048	0.0489
$45^\circ$	0.0075	0.0077	0.0071	0.0076	0.0413	0.042	0.041	0.0420
$60^\circ$	0.0054	0.0053	0.0049	0.0054	0.0324	0.032	0.032	0.0322
$75^\circ$	0.0012	0.0023	0.0010	0.0017	0.0181	0.018	0.018	0.0180
$90^\circ$	0.0003	0.0000	0.0003	0.0000	0.0000	0.000	0.000	0.0000

### Square plate with round hole and two cracks

**Fig. 11:**
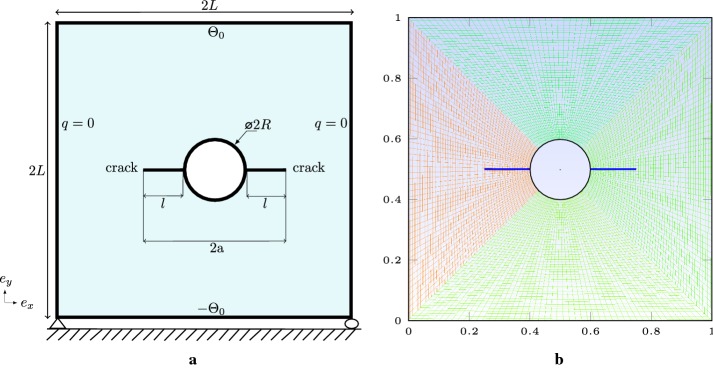
Square plate with round hole and two cracks: **a** boundary conditions representation, **b** structured mesh used

In this example, we consider the case of a square plate with a hole and two cracks. The objective is: firstly, to show the influence of the presence of a hole, due to a manufacturing defect or willingly introduced into the material, on the stress intensity factors. Secondly, to examine the influence of radius of the fixed enriched zone on the SIFs. Thirdly, to show the influence of the characteristic length (*h*) on the convergence of SIFs when *h* goes to zero. The dimensions of the domain are chosen such $L=0.5\;\mathrm{m}$, the hole is placed in the center of the plate defined by the radius *R* Fig. 11a. The two cracks are defined at the two ends, right and left, of the hole are centered (right and left cracks), with a length *l*. Half-length of the apparent crack is defined by $a=l+R$. The bottom left corner is clamped and displacements along $e_{y}$-axis is fixed. The heat flux is zero at the right and left edges, an imposed temperature of $\pm \Theta _{0}$ are defined at the top and bottom sides, such $\Theta _{0}=10\;^\circ \mathrm{C}$.

**Fig. 12:**
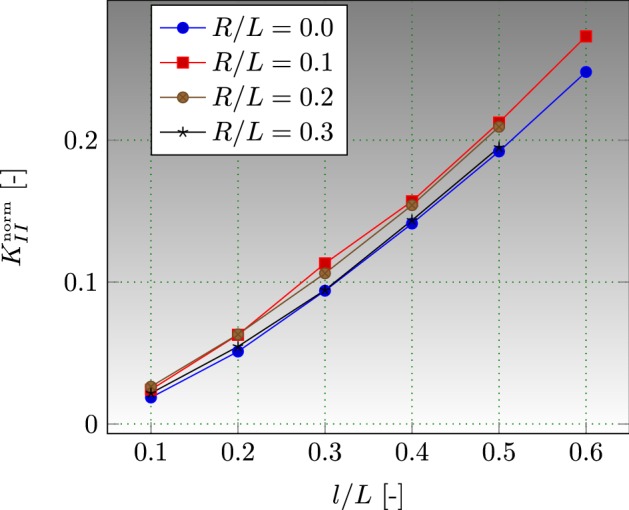
Normalized SIFs for a square plate with round hole, various *R* / *L* and *l* / *L*

We investigate the influence of various fractions of hole size *R* / *L* and cracks sizes *l* / *L* on the SIFs computations. We choose a set of *R* / *L* resp., *l* / *L* between 0.0 and 0.3, resp., 0.1 and 0.6 with a jump of 0.1. The structured mesh is used Fig. 11b, such the characteristic length is $0.011\;\mathrm{m}$. It is worth noting that when *R* / *L* and *l* / *L* become too small, we refine sufficiently close to the two cracks to ensure a good approximation of the SIFs for the J-integral domain. The stress intensity factors are dominated by mode-II; we normalize it by $\alpha \Theta _{0}E\sqrt{W}$ which gives $K_{II}^{\mathrm{norm}}$. The normalized SIFs for several choices of the two ratios are illustrated in Fig. 12. Results obtained by the XFEM-f.a. are close to those given by Prasad et al. [44]. Complete results of the two cracks are given in Appendix: Table 10.

Influence of the radius of the fixed enriched area on the SIFs computation is inspected for several values of $E_{R}$. A typical case are chosen for a hole with $R/L=0.1$ and $l/L=\{0.5,0.6\}$ for both left and right cracks, Table 6. It can be seen that there is no significant difference in the computation of SIFs, for both left and right cracks, with respect to the choice of the radius value of the enrichment disk. A relatively large radius related (and independently) to the characteristic length of the mesh is desirable. As mentioned before, when the two ratios have become small, we tend to refine the mesh in the vicinity of the crack. Therefore, the selection of radius must be adapted to the size of the crack and the characteristic length.

**Table 6:** Influence of enrichment radii on the computation of SIFs for square plate with round hole and two cracks, $R/L=0.1$

$\frac{\varvec{l}}{\varvec{L}}$	**Normalized SIFs**	**Left crack**			**Right crack**		
		$\varvec{E}_{\varvec{R}}$			$\varvec{E}_{\varvec{R}}$		
		**0.2**	**0.15**	**0.1**	**0.2**	**0.15**	**0.1**
0.5	$K_{II}^{\mathrm{norm}}$	0.219244	0.219256	0.219357	$-$ 0.219244	$-$ 0.219256	$-$ 0.219357
0.6	$K_{II}^{\mathrm{norm}}$	0.273149	0.273162	0.273056	$-$ 0.273149	$-$ 0.273162	$-$ 0.273056

**Fig. 13:**
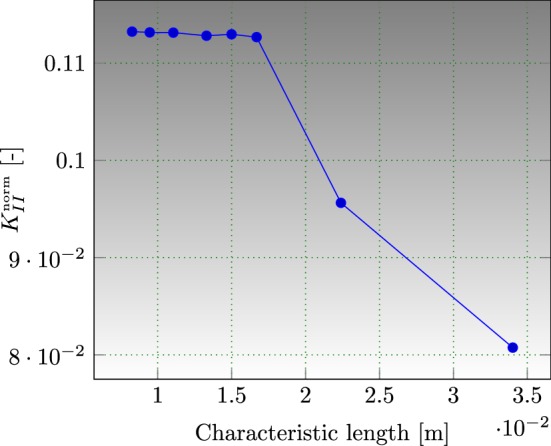
Convergence of SIFs computation, square plate with round hole, $R/L=0.1$ and $l/L=0.3$

Convergence of SIFs computation has been demonstrated with respect to the size of the mesh, by comparing between a range of a coarse mesh and a sufficiently finer one. As illustrated in Fig. 13, XFEM-f.a. guarantees a significant convergence of the thermo-mechanical model and the SIFs computation.

**Fig. 14:**
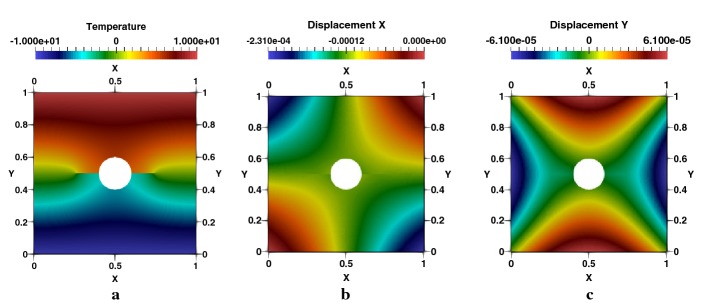
Square plate with round hole, $R/L=0.2$, $l/L=0.3$: **a** temperature, **b** $e_{x}$-displacement, **c** $e_{y}$-displacement

**Fig. 15:**
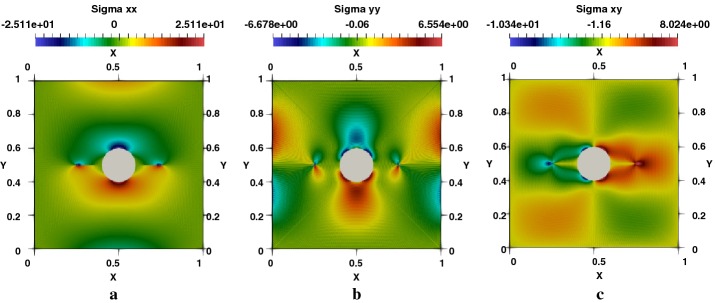
Square plate with round hole, $R/L=0.2$, $l/L=0.3$: **a** $\sigma _{xx}$-stress, **b** $\sigma _{yy}$-stress, **c** $\sigma _{xy}$-stress

Distribution of the temperature generated by the effect of the crack and hole is presented in Fig. 14a. Displacements in $e_{x}$ and $e_{y}$ Directions are given respectively in Fig. 14b, c. We note an important concentration of the stress in the two crack-tips and around the perimeter of the hole in the vertical direction, as showed respectively in Fig. 15a–c for $\sigma _{xx}$, $\sigma _{yy}$ and $\sigma _{xy}$. The heat flux in $e_{x}$ and $e_{y}$ directions are figured respectively in 16a–c represents the spatial distribution of the flux lines that bypasses both the cracks and the hole.

**Fig. 16:**
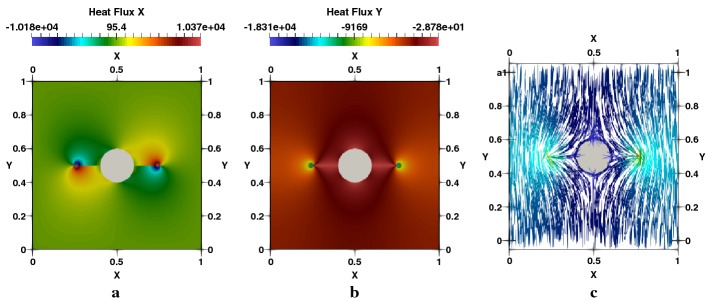
Square plate with round hole $R/L=0.2$, $l/L=0.3$: **a** $q_{x}$-heat flux, **b** $q_{y}$-heat flux, **c** flux lines

### Edge cracked strip under thermal loading

**Fig. 17:**
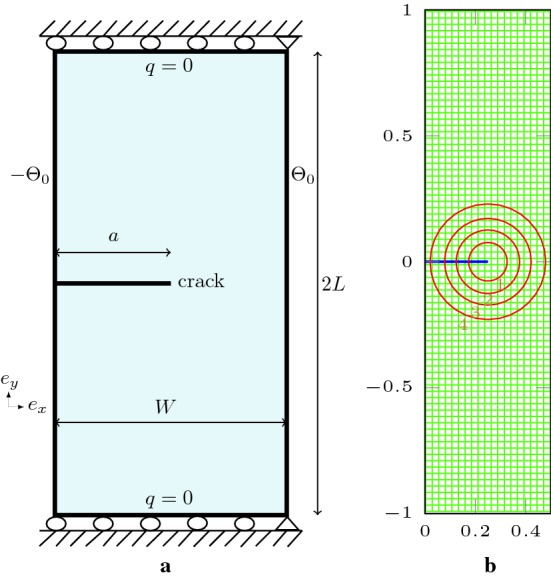
Edge cracked strip under pure thermal load: **a** geometry and crack growth boundary conditions, **b** structured mesh used

We analyze in this example a case of the thermo-mechanical crack propagation of an edge cracked plate subjected to a pure thermal load in first, governed by mode-I, and under transient-thermal load in the second case. A rectangular plate of $W\times 2L$, with the width $W=0.5\;\mathrm{m}$ and height $L=1.0\;\mathrm{m}$, is assumed with an initial edge crack $a=0.25\;\mathrm{m}$ at the middle of left edge, $\Gamma _{c}=[0,0.25]\times \{0\}$. Displacements along the $e_{y}$-axis are fixed at the bottom, and top edges excepted both the bottom and top right corners where the plate is embedded. The two top and bottom sides are considered insulated, i.e., the heat flux *q* is zero; a prescribed temperatures of $\pm \Theta _{0}$ are imposed at the right and left boards, such $\Theta _{0}=10\;^\circ$C in Fig. 17a. Crack geometry and the structured rectangular uniform mesh of 30 elements upon the width and 120 across the height is shown in Fig. 17b. Material properties in Table 1 is considered to illustrate the profiles of temperature, displacements, stress and heat flux for a specific choice of material. Young’s modulus $\overline{E}=10^{3}E$, while the computation of SIFs are normalized by dividing $K_{I}$ by $\sigma _{\Theta _{0}}\sqrt{\pi a}$, with $\sigma _{\Theta _{0}}=(E/(1-\nu ))\alpha \Theta _{0}$ the stress at the right bord of the uncracked strip. This definition gives $K_{I}^{\mathrm{norm}}$ introduced in the transient-load case. The temperature distribution is linear in the $e_{x}$ direction, $\Theta =(\frac{2\Theta _{0}}{W})x$. Disks radii $E_{R}$ is taken equal to $0.1\;\mathrm{m}$.

#### Thermal transient loading

**Fig. 18:**
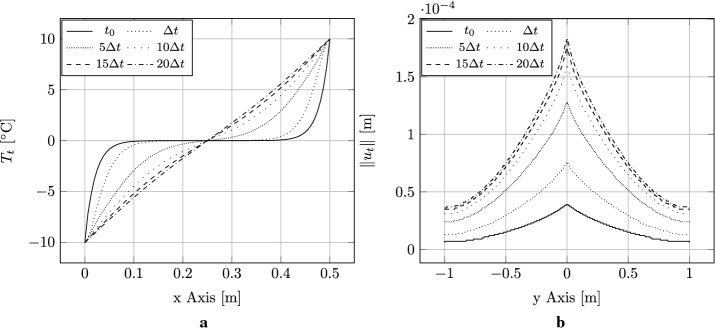
Edge cracked strip under transient thermal load: **a** temperature field, cut over [(0,0);($\frac{1}{2}$,0)] line, **b** euclidean norm of displacement, cut over the line [($\frac{1}{5}$,−1);($\frac{1}{5}$,1)]

During this application, a gradual transient load is taken into account, keeping the same configuration of physical domain presented above. Example is performed for the same material parameters, with a solid density of $\rho =2.7$ [kg/m$^3$] and a heat capacity of $c=921$ [(W.s)/($^\circ$C.kg)]. The end time $\overline{T}_{f}$ is taken equal to unity with a uniform, constant time step with a maximum number of increments of 30. The temperature distribution is linear in space and in time horizontally and remains uniform along the $e_{y}$ axis, so a typical cut over the line-section $\{x\in \Omega \setminus \Gamma _{c}\;;\;y=0\}$ is presented for different increments until reaching the thermal equilibrium, as shown in Fig. 18a. On the left border, the plate tends to expand. Additionally, the displacement is fixed along $e_{y}$-axis throughout the top and bottom sides; this generates a significant displacement, with respect to the pseudo-time, of the upper crack surface towards $e_{y}$ and symmetrical displacement of the lower crack surface towards $-e_{y}$. This behavior leads to a gradual opening of the crack with the continuous transient load until the achievement of the equilibrium state. Figure 18b depict the Euclidean norm of displacement combining the horizontal and vertical one over the line-section $\{y\in \Omega \setminus \Gamma _{c};\;x=\frac{1}{5}\}$. The stress, with respect to the pseudo-time, also becomes important near the crack tip after the incremental thermal loading.

**Fig. 19:**
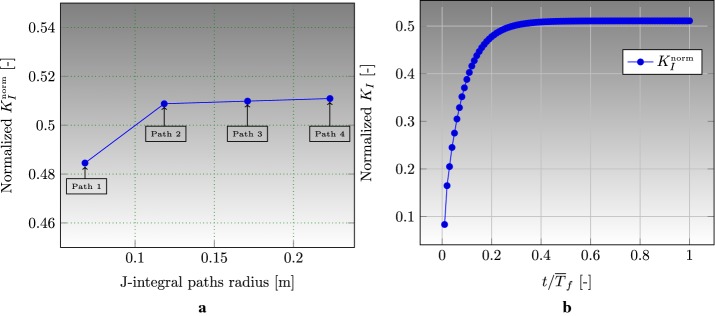
Edge cracked strip under transient thermal load: **a** normalized $K_{I}$ versus J-integral paths, at final time $\overline{T}_{f}$, **b** normalized $K_{I}$ over time

A reassessment of the path-dependence of the J-integral computation, with respect to the radius domain, over several paths is treated for this case in Fig. 19a. Selected paths are indexed using red lines, by ‘1’ to ‘4’ in Fig. 17b. The curve of normalized SIFs at the final equilibrium step is drawing. We note that the computation of SIFs evidently converges for a large choice of the path radius. The computed normalized ’transient’ $K_{I}^{\mathrm{norm}}(t)$ stress intensity factor is plotted in Fig. 19b; the profile progresses with a positive slope and hold steady from a value of $\frac{t}{\overline{T}_{f}}\geqslant 0.3$. This stage implicitly interprets, as another way, that the equilibrium state is reached by a post-XFEM-f.a. quantity, $K_{I}$.

**Fig. 20:**
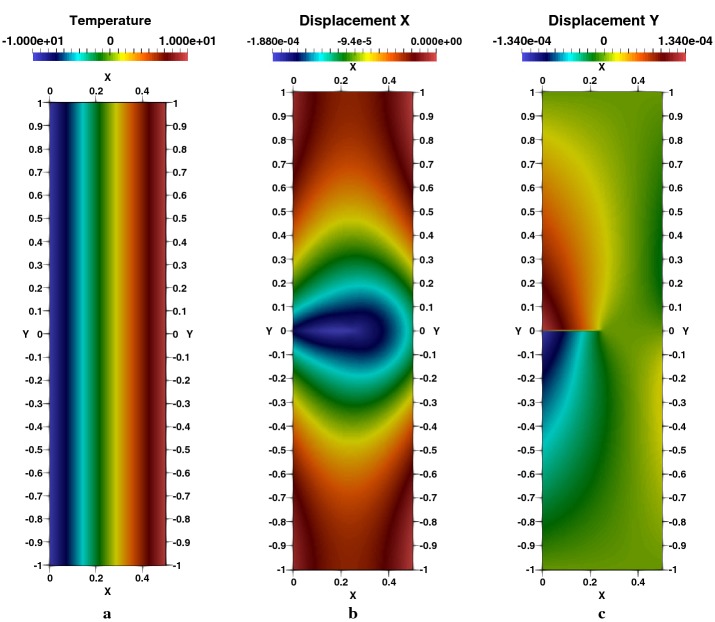
Edge cracked strip under transient thermal load at final time $\overline{T}_{f}$: **a** temperature, **b** $e_{x}$-displacement, **c** $e_{y}$-displacement

Thermal equilibrium subsequently leads to a mechanical one; its primary phase starts from $\frac{t}{\overline{T}_{f}}\in [20\Delta t,1]$. Figure 20a–c represent respectively the distribution of temperature, $e_{x}$-displacement and $e_{y}$-displacement at the final stage.

#### Crack growth

**Fig. 21:**
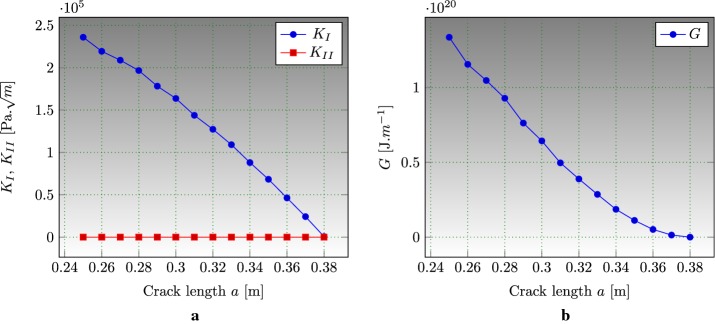
Crack growth in edge cracked strip: **a** stress intensity factors $K_{I}$ and $K_{II}$, **b** energy release rate *G*

**Fig. 22:**
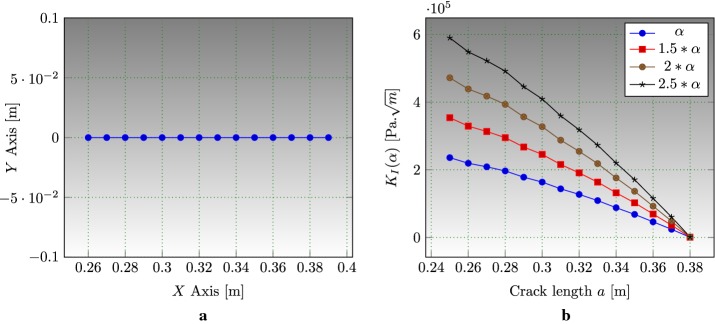
Crack growth in edge cracked strip: **a** crack path, **b** influence of expansion coefficient on SIFs, $K_{I}$

This section shows an application of XFEM-f.a. in the thermo-mechanical growth of an edge crack governed by mode-I. The configuration is taken with the same considerations and initial crack as mentioned above. Crack growth is monitoring using hoop stress, by introducing an additional virtual crack extension (VCE) based on VCE-method Hellen [55], Millwater et al. [56] after each equilibrium step. The direction of discrete crack propagation is determined by the orientation in which the maximum energy is released from the system. Crack propagation was simulated for a total of 14 steps, with each step size of length $\Delta a = 0.01\;\mathrm{m}$. Convergence of SIFs is proved by Fig. 13; simply choose a sufficiently finer mesh to guarantee an optimal convergence, here characteristic length is $0.01\;\mathrm{m}$. Determination of crack path is tested for several crack magnitudes $\Delta a$, $2\Delta a$, $3\Delta a$ and $5\Delta a$ and converges to the same path. Stress intensity factors $K_{I}$, $K_{II}$ in Fig. 21a show a crack growth driven by mode-I, $K_{II}$ keeps a zero value for all steps. Stability of crack growth is illustrated by Fig. 21b, where the variation of energy release rate *G* remains negative for the total of increments. Crack progresses in a parallel direction to $e_{x}$ and at $y=0$, Fig. 22a, which means again the dominated character of $K_{I}$. Figure 22b demonstrates that the thermal expansion coefficient obviously influences stress intensity factors. The average slope value of SIFs profiles in growth is inversely proportional to the coefficient of thermal expansion. The set of SIFs profiles corresponding to the different values of the expansion coefficient keep the same pace and converge systematically to the same point of $K_{I}=0$. This result explains that for any choice of $\alpha$, for any other material properties, the system converges towards the same end-growth point.

### Rectangular plate with two circular holes and multiple cracks

**Fig. 23:**
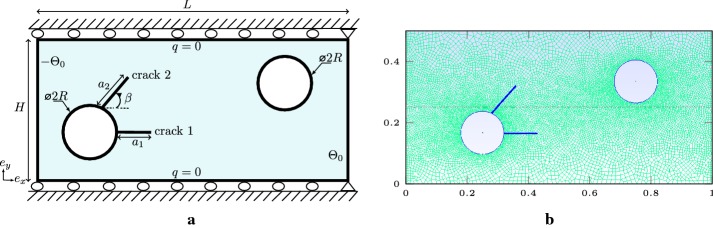
Plate with two circular holes and multiple cracks: **a** geometry and boundary conditions for crack growth, **b** computational mesh used

A numerical example of a $H\times L$ rectangular plate, containing two circular holes and two cracks was designed. Physical and numerical framework ensuring a thermo-mechanical crack propagation is well defined and sketched in Fig. 23a. The width $H=0.5\;\mathrm{m}$, the length $L=1.0\;\mathrm{m}$ and the radius of each circle is $R=0.07\;\mathrm{m}$. Initial cracks are set to start from the limit of the left hole; the first crack termed ‘crack 1’ is a straight crack with a length $a_{1}=0.05\;\mathrm{m}$, the second crack termed ‘crack 2’ is an inclined crack with an angle $\beta =60^\circ$ and a length $a_{2}=0.1\;\mathrm{m}$. Displacements of the specimen are fixed vertically throughout the upper and lower part, excluding the two right corners which are embedded. The right edge is cold at $-\Theta _{0}$ and hot on the left one at $\Theta _{0}$ temperature, where $\Theta _{0}=20\;^\circ \mathrm{C}$. The plate is completely insulated on the top and bottom borders. The computational domain is outlined in Fig. 23b, characteristic length of the mesh used is $0.012\;\mathrm{m}$; we refine a bit more throughout the borders of the two circles. Material properties are defined in the Table 1, with the consideration of Young’s modulus $\overline{E}$ and a specific choice of thermal expansion coefficient $\overline{\alpha }=10\alpha$. The radius $E_{R}$ of disks related to each crack tip is taken uniform and equal to 0.1m. No reference found in the literature dealing with this crack growth example for comparison.

**Fig. 24:**
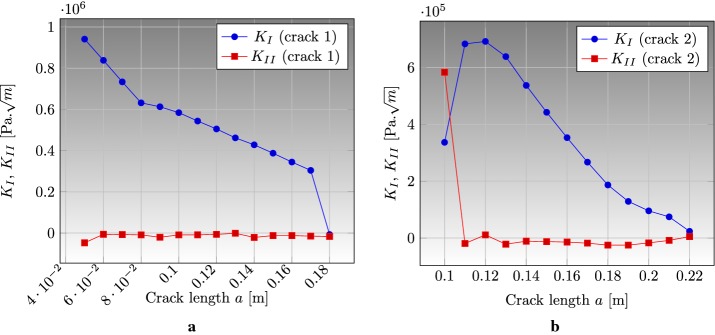
Crack growth in plate with two circular holes and multiple cracks: stress intensity factors $K_{I}$ and $K_{II}$; **a** crack 1, **b** crack 2

The configuration of specimen defined in this case, presenting two material defects (holes) and multiple cracks, allows simulating a thermo-mechanical crack growth by XFEM-f.a. driven by mixed mode. Similarly, the VCE method is assumed for the progression of cracks. Furthermore, considering the two cracks on the border of the left hole is not an arbitrary choice. The idea is to identify the zone which has an important stress concentration enabling a progression ’opening’. Cracks move forward simultaneously with a combined propagation criterion for both of them. The growth was simulated for a total of 13 extension steps, with each step size of length $\Delta a= 0.01\;\mathrm{m}$. Several crack magnitudes and sufficient characteristic lengths of the mesh are chosen and converge to the appropriate crack path. Stress intensity factors in mode-I and II, $K_{I}$ and $K_{II}$, of the two cracks ’1’ and ’2’ are presented respectively in Fig. 24a, b. Crack 1 is driven by a dominated mode-I from the beginning with a range of $\sim 10^{6}$ that is sufficient to preserve a progressive growth. Whereas, the intensity of the driven magnitude at the vicinity of the initial crack-tip 2 is 10 times less than the crack-tip 1 and 2 times less than the intensity required to evolve the crack 2. This case brings to a crack ’initiation’ controlled by both mode-I and II for the first extension and described by a drop, of $\sim 6$ times less, of $K_{II}$ and rise, of $\sim 2$ times more, of $K_{I}$.

**Fig. 25:**
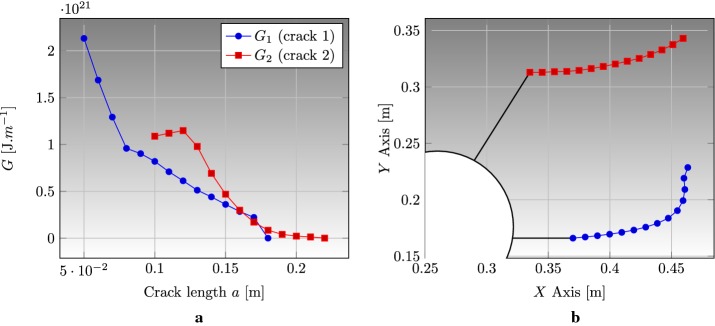
Crack growth in plate with two circular holes and multiple cracks: **a** energy release rate $G_{.}$, **b** cracks path

Energy release rate $G_{1}$ of crack 1 and $G_{2}$ of crack 2, Fig. 25a, keep the same behavior as the stress intensity factors. The $G_{1}$ remains stable throughout the incremental progression; while crack 2 seeks to reach local stability for the first two extensions by getting the required energy to progress crack and subsequently maintain the stability. Crack paths are depicted in Fig. 25b including a representation of the initial preexisting cracks and the left round hole.

**Fig. 26:**
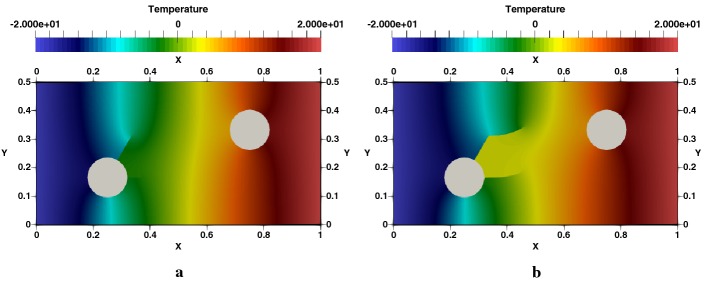
Crack growth in plate with two circular holes and multiple cracks: Temperature profile; **a** initial state, **b** 13$\Delta a$

The crack arrest is taken for a state related to the configuration defined by $13\Delta a$. This represents the state where the crack 1 develops locally an important compression at the crack surfaces; it produced by the new material configuration caused by the displacement of the lower surface of crack 2 along the $-e_{y}$ axis. The specimen is thermally loaded throughout the process of crack propagation. Consequently, the temperature profile is affected by the new configuration of the cracks which causes an incremental change in the spatial redistribution of the temperature; the initial state and the final one are shown respectively in Fig. 26a, b.

**Fig. 27:**
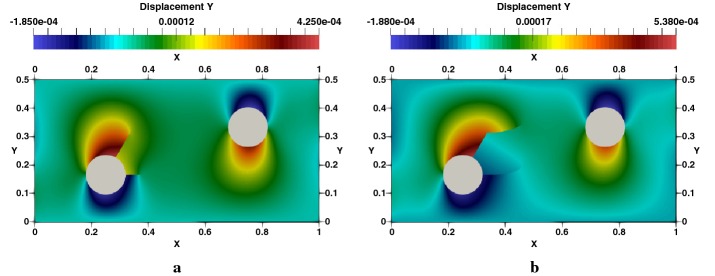
Crack growth in plate with two circular holes and multiple cracks: $e_{y}$-displacement; **a** initial state, **b** 13$\Delta a$

Displacements are more pronounced over $e_{y}$ direction; Fig. 27a, b stand for respectively $e_{y}$-displacement at initial state and at $13\Delta a$ state.

**Fig. 28:**
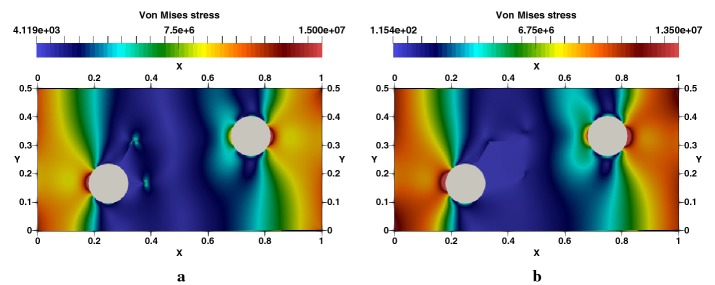
Crack growth in plate with two circular holes and multiple cracks: von Mises stress—$\sigma _{\tiny \text{ VM }}$; **a** initial state, **b** 13$\Delta a$

Stress combining the information of different directional stresses is given by the von Mises stress, $\sigma _{{\tiny \text{ VM }}}$, at the initial state in Fig. 28a and at the final one in Fig. 28b. Spatial redistribution of $\sigma _{{\tiny \text{ VM }}}$, induced by the temperature profile, varies with the crack growth. Stress becomes maximal near the crack tips and over the outer borders of the two holes at the initial state; it increases for the holes and decreases slightly at the tip points thanks to the release of energy caused by the crack progression.

## Conclusions

A new thermo-mechanical crack propagation model in a cracked body was presented which can be applied, for instance, to ensure the safety of structures subjected to thermal loading. The developed geometrical eXtended finite element method was successfully applied to model crack growth and achieving the expected optimal rate of convergence by confirming the benefit of the fixed enrichment area approach on the computation of stress intensity factor profile. Numerical development and various matrices in full coupling were presented for each sub-problems, mechanical and thermal, and for the full coupled XFEM part. The criteria for crack growth, as well as for the direction of the virtual crack extension are described, and their performance in the context of the XFEM is discussed. From three examples, various benchmarks result in a cracked domain are examined and validated from the existing results in the literature. The robustness and the accuracy of the model implementation to extract the thermo-mechanical responses and to compute the associated stress intensity factors for stationary crack, with and without holes, as well as the effect of crack length and hole position on the SIFs are proved. Furthermore, a quasi-transient load example governed by mode-I is presented and the contribution of this loading on the profile of the SIFs until reaching thermal equilibrium is analyzed. Finally, an example of multiple mixed-mode cracks growth and multiple holes that may be present as small flaws in the material manufacturing stage is examined; only the limiting cases of stable crack are discussed. When the heat flow is distributed by the presence of the cracks, we observe a high local intensification of thermal gradients followed by an intensification of thermo-mechanical stress around them, which may lead to the crack growth or inevitable collapse of the structure.

As outlook of future works, possible improvement of this study can be made by taking into consideration the mechanical contact aspect between the crack surfaces; this will be important to extend to study of the last example to simulate the complete process of growth. Another point can be viewed by holding the crack propagation in the overall dynamic of the whole problem and admitting a crack-pseudo-time-dependant; which make it possible to control the evolution of the crack with the transient loading. This case requires a sophisticated treatment of the stiffness matrix; *K* needs to be evaluated at two different times for two different configurations of the crack, $K_{i}$ and $K_{i+1}$.

## Appendix: Complete stress intensity factors tables

See Tables 7, 8, 9, and 10

**Table 7:** Normalized SIFs for centred crack in a square plate, various *a* / *W*

$\frac{\varvec{a}}{\varvec{W}}$	**Normalized SIFs**	**Left tip**		**Right tip**			
		**Present work**	[27, 44, 54]	**Present work**	[54]	[44]	[27]
0.1	$K_{I}^{\mathrm{norm}}$	0.000000	–	0.000000	–	–	–
	$K_{II}^{\mathrm{norm}}$	$-$ 0.018191	–	0.018191	0.021	0.018	0.019
0.2	$K_{I}^{\mathrm{norm}}$	0.000000	–	0.000000	–	–	–
	$K_{II}^{\mathrm{norm}}$	$-$ 0.053515	–	0.053515	0.053	0.054	0.054
0.3	$K_{I}^{\mathrm{norm}}$	0.000000	–	0.000000	–	–	–
	$K_{II}^{\mathrm{norm}}$	$-$ 0.096664	–	0.096664	0.094	0.095	0.096
0.4	$K_{I}^{\mathrm{norm}}$	0.000000	–	0.000000	–	–	–
	$K_{II}^{\mathrm{norm}}$	$-$ 0.141209	–	0.141209	0.141	0.141	0.141
0.5	$K_{I}^{\mathrm{norm}}$	0.000000	–	0.000000	–	–	–
	$K_{II}^{\mathrm{norm}}$	$-$ 0.192025	–	0.192025	0.188	0.190	0.191
0.6	$K_{I}^{\mathrm{norm}}$	0.000000	–	0.000000	–	–	–
	$K_{II}^{\mathrm{norm}}$	$-$ 0.248057	–	0.248057	0.247	0.243	0.245

**Table 8:** Normalized SIFs for slope crack at both tips, $\beta =30^\circ$ and various *a* / *W*

$\frac{\varvec{a}}{\varvec{W}}$	**Normalized SIFs**	**Left tip**				**Right tip**			
		**Present work**	[54]	[44]	[27]	**Present work**	[54]	[44]	[27]
0.2	$K_{I}^{\mathrm{norm}}$	$-$ 0.002181	–	–	–	0.002181	0.002	0.002	0.0020
	$K_{II}^{\mathrm{norm}}$	0.030191	0.030	0.030	0.0302	$-$ 0.030191	–	–	–
0.3	$K_{I}^{\mathrm{norm}}$	$-$ 0.006959	–	–	–	0.006956	0.008	0.006	0.0068
	$K_{II}^{\mathrm{norm}}$	0.048436	0.048	0.048	0.0489	$-$ 0.048435	–	–	–
0.4	$K_{I}^{\mathrm{norm}}$	$-$ 0.015213	–	–	–	0.015212	0.015	0.014	0.0149
	$K_{II}^{\mathrm{norm}}$	0.064008	0.064	0.064	0.0650	$-$ 0.064010	–	–	–
0.5	$K_{I}^{\mathrm{norm}}$	$-$ 0.026998	–	–	–	0.026999	0.027	0.026	0.0265
	$K_{II}^{\mathrm{norm}}$	0.077393	0.076	0.076	0.0774	$-$ 0.077393	–	–	–
0.6	$K_{I}^{\mathrm{norm}}$	$-$ 0.040855	–	–	–	0.040859	0.041	0.040	0.0407
	$K_{II}^{\mathrm{norm}}$	0.087247	0.086	0.087	0.0878	$-$ 0.087248	–	–	–

**Table 9:** Normalized SIFs for slope crack at both tips, $a/W=0.3$ and various $\beta$

$\varvec{\beta }$	**Normalized SIFs**	**Left tip**				**Right tip**			
		**Present work**	[54]	[44]	[27]	**Present work**	[54]	[44]	[27]
$0^\circ$	$K_{I}^{\mathrm{norm}}$	0.000000	0.0000	0.0000	0.0000	0.000000	–	–	–
	$K_{II}^{\mathrm{norm}}$	$-$ 0.054852	–	–	–	0.054852	0.054	0.054	0.0546
$15^\circ$	$K_{I}^{\mathrm{norm}}$	$-$ 0.003698	–	–	–	0.003699	0.0038	0.0036	0.0038
	$K_{II}^{\mathrm{norm}}$	0.053371	0.054	0.054	0.0533	$-$ 0.053370	–	–	–
$30^\circ$	$K_{I}^{\mathrm{norm}}$	$-$ 0.006959	–	–	–	0.006956	0.0071	0.0064	0.0068
	$K_{II}^{\mathrm{norm}}$	0.048436	0.048	0.048	0.0489	$-$ 0.048435	–	–	–
$45^\circ$	$K_{I}^{\mathrm{norm}}$	$-$ 0.007509	–	–	–	0.007508	0.0077	0.0071	0.0076
	$K_{II}^{\mathrm{norm}}$	0.041381	0.042	0.041	0.0420	$-$ 0.041382	–	–	–
$60^\circ$	$K_{I}^{\mathrm{norm}}$	$-$ 0.005460	–	–	–	0.005460	0.0053	0.0049	0.0054
	$K_{II}^{\mathrm{norm}}$	0.032463	0.032	0.032	0.0322	$-$ 0.032463	–	–	–
$75^\circ$	$K_{I}^{\mathrm{norm}}$	$-$ 0.001284	–	–	–	0.001284	0.0023	0.0010	0.0017
	$K_{II}^{\mathrm{norm}}$	0.018197	0.018	0.018	0.0180	$-$ 0.018197	–	–	–
$90^\circ$	$K_{I}^{\mathrm{norm}}$	0.000394	0.0000	0.0003	0.0000	$-$ 0.000394	–	–	–
	$K_{II}^{\mathrm{norm}}$	0.000000	–	–	–	0.000000	0.000	0.000	0.0000

**Table 10:** Normalized SIFs for square plate with hole and two cracks, various *l* / *L* and *R* / *L* (Left-crack := Lc and Right-crack := Rc)

$\varvec{l/L}$	**Normalized SIFs**	**R/L=0.0**		**R/L=0.1**		**R/L=0.2**		**R/L=0.3**	
		**Lc**	**Rc**	**Lc**	**Rc**	**Lc**	**Rc**	**Lc**	**Rc**
0.1	$K_{I}^{\mathrm{norm}}$	0.000000	0.000000	0.000000	0.000000	0.000000	0.000000	0.000000	0.000000
	$K_{II}^{\mathrm{norm}}$	$-$ 0.018643	0.018643	0.024200	$-$ 0.024200	0.026359	$-$ 0.026359	0.021674	$-$ 0.021674
0.2	$K_{I}^{\mathrm{norm}}$	0.000000	0.000000	0.000000	0.000000	0.000000	0.000000	0.000000	0.000000
	$K_{II}^{\mathrm{norm}}$	$-$ 0.051048	0.051048	0.062837	$-$ 0.062837	0.063162	$-$ 0.063162	0.054608	$-$ 0.054608
0.3	$K_{I}^{\mathrm{norm}}$	0.000000	0.000000	0.000000	0.000000	0.000000	0.000000	0.000000	0.000000
	$K_{II}^{\mathrm{norm}}$	$-$ 0.093898	0.093898	0.113145	$-$ 0.113145	0.106177	$-$ 0.106177	0.094392	$-$ 0.094392
0.4	$K_{I}^{\mathrm{norm}}$	0.000000	0.000000	0.000000	0.000000	0.000000	0.000000	0.000000	0.000000
	$K_{II}^{\mathrm{norm}}$	$-$ 0.141210	0.141210	0.156955	$-$ 0.156955	0.154166	$-$ 0.154166	0.143558	$-$ 0.143558
0.5	$K_{I}^{\mathrm{norm}}$	0.000000	0.000000	0.000000	0.000000	0.000000	0.000000	0.000000	0.000000
	$K_{II}^{\mathrm{norm}}$	$-$ 0.192025	0.192025	0.219244	$-$ 0.219244	0.209395	$-$ 0.209395	0.194878	$-$ 0.194878
0.6	$K_{I}^{\mathrm{norm}}$	0.000000	0.000000	0.000000	0.000000	–	–	–	–
	$K_{II}^{\mathrm{norm}}$	$-$ 0.248057	0.248057	0.273149	$-$ 0.273149	–	–	–	–
